# Morphology and structure of *Homo erectus* humeri from Zhoukoudian, Locality 1

**DOI:** 10.7717/peerj.4279

**Published:** 2018-01-19

**Authors:** Song Xing, Kristian J. Carlson, Pianpian Wei, Jianing He, Wu Liu

**Affiliations:** 1 Key Laboratory of Vertebrate Evolution and Human Origins of Chinese Academy of Sciences, Institute of Vertebrate Paleontology and Paleoanthropology, Chinese Academy of Sciences, Beijing, China; 2 Department of Integrative Anatomical Sciences, Keck School of Medicine, University of Southern California, Los Angeles, CA, USA; 3 Evolutionary Studies Institute, University of the Witwatersrand, Johannesburg, South Africa; 4 MOE Key Laboratory of Contemporary Anthropology, Collaborative Innovation Center for Genetics and Development, School of Life Sciences, Fudan University, Shanghai, China; 5 School of Archaeology and Museology, Peking University, Beijing, China

**Keywords:** East Asia, Pleistocene, Hominin, Upper limb, Diaphyseal robusticity

## Abstract

**Background:**

Regional diversity in the morphology of the *H. erectus* postcranium is not broadly documented, in part, because of the paucity of Asian sites preserving postcranial fossils. Yet, such an understanding of the initial hominin taxon to spread throughout multiple regions of the world is fundamental to documenting the adaptive responses to selective forces operating during this period of human evolution.

**Methods:**

The current study reports the first humeral rigidity and strength properties of East Asian *H. erectus* and places its diaphyseal robusticity into broader regional and temporal contexts. We estimate true cross-sectional properties of Zhoukoudian Humerus II and quantify new diaphyseal properties of Humerus III using high resolution computed tomography. Comparative data for African *H. erectus* and Eurasian Late Pleistocene *H. sapiens* were assembled, and new data were generated from two modern Chinese populations.

**Results:**

Differences between East Asian and African *H. erectus* were inconsistently expressed in humeral cortical thickness. In contrast, East Asian *H. erectus* appears to exhibit greater humeral robusticity compared to African *H. erectus* when standardizing diaphyseal properties by the product of estimated body mass and humeral length. East Asian *H. erectus* humeri typically differed less in standardized properties from those of side-matched Late Pleistocene hominins (e.g., Neanderthals and more recent Upper Paleolithic modern humans) than did African *H. erectus*, and often fell in the lower range of Late Pleistocene humeral rigidity or strength properties.

**Discussion:**

Quantitative comparisons indicate that regional variability in humeral midshaft robusticity may characterize *H. erectus* to a greater extent than presently recognized. This may suggest a temporal difference within *H. erectus*, or possibly different ecogeographical trends and/or upper limb loading patterns across the taxon. Both discovery and analysis of more adult *H. erectus* humeri are critical to further evaluating and potentially distinguishing between these possibilities.

## Introduction

*Homo erectus* has been portrayed as a geochronologically persistent taxon encompassing a great deal of regional diversity over its evolutionary history (Antón, 2003). The initial appearance of *H. erectus* in the hominin fossil record is approximately 1.9 Ma from Koobi Fora, Kenya, while the late persistence documented in Southeast Asia (i.e., Ngandong at 80 Ka) is unmatched elsewhere (Dubois, 1894; Dubois, 1936; Black, 1930; Black, 1933; Von Koenigswald, 1936; Von Koenigswald, 1940; Von Koenigswald, 1951; Weidenreich, 1938; Weidenreich, 1941; Weidenreich, 1943; Woo, 1964; Woo, 1966; Chiu et al., 1973; Hu, 1973; Jacob, 1973; Santa Luca, 1980; Wu & Dong, 1982; Wu & Poirier, 1995; Swisher et al., 1996; Antón, 2003; Kaifu, Aziz & Baba, 2005a; Kaifu et al., 2005b; Liu, Zhang & Wu, 2005; Zhu et al., 2008; Zaim et al., 2011). Characterization of the taxon as regionally diverse emphasizes craniodental features (Rightmire, 1998; Antón, 2003; Kaifu, Aziz & Baba, 2005a; Kaifu et al., 2005b; Baab, 2008; Lordkipanidze et al., 2013; Antón et al., 2016) in focusing on hominin systematics (Howells, 1980; Stringer, 1984; Rightmire, 1993; Wood, 1994; Antón, 2002; Antón, 2003) and feeding behaviour (Ungar, Grine & Teaford, 2006). By comparison, emphasis on *H. erectus* postcrania is less frequent when framing *H. erectus* diversity (Ruff, 2008; Pontzer et al., 2010; Puymerail et al., 2012; Ruff et al., 2015b).

Relative scant attention given to regional diversity in *H. erectus* postcranial fossils, in part, is a function of the paucity of Asian sites preserving postcranial fossils (Antón, 2003); upper limb elements of East Asian hominins, such as humeri, have been recovered only from Zhoukoudian (see Weidenreich, 1941). As a result, current depictions of *H. erectus* postcranial morphology draw heavily from the more abundant African, Georgian, and to a lesser extent Southeast Asian, *H. erectus* fossils (e.g., Ruff, 2008; Pontzer et al., 2010; Puymerail et al., 2012; Ruff et al., 2015b). This work traditionally emphasizes the relatively complete immature skeleton, KNM-WT 15000 (Walker & Leakey, 1993), a partial adult skeleton from Kenya, KNM-ER 1808 (Walker, Zimmerman & Leakey, 1982; Leakey & Walker, 1985), and sets of postcranial fossils from multiple individuals represented at Dmanisi (Lordkipanidze et al., 2007; Pontzer et al., 2010). Characterization of postcranial regional diversity in *H. erectus*, therefore, would benefit from expanding upon these efforts to include East Asian fossils. The aim of the present study is to broaden the current understanding of regional diversity in *H. erectus* by conducting the first quantitative investigation of diaphyseal strength properties in East Asian *H. erectus* humeri.

Cross-sectional geometric properties of long bone diaphyses provide a useful means of inferring activity patterns in past populations (Ruff et al., 1993; Trinkaus, Churchill & Ruff, 1994; Trinkaus, 1997; Stock, 2006; Carlson, Grine & Pearson, 2007; Ruff, 2008; Carlson & Marchi, 2014; Ruff & Larsen, 2014, and references therein; Sládek et al., 2016), although these inferences are not always straightforward (Pearson & Lieberman, 2004; Ruff, Holt & Trinkaus, 2006; Wallace et al., 2012). Relatively recent temporal declines in humeral diaphyseal robusticity from archaic *H. sapiens* to modern *H. sapiens* have been well-documented across Eurasia and Africa (Ruff et al., 1993; Trinkaus, Churchill & Ruff, 1994; Trinkaus, 1997). Likewise, marked bilateral asymmetry in humeral strength appears to have emerged in, and been more consistently expressed by, Eurasian Late Pleistocene hominins compared to those of the Holocene, which is when presumed activity-related reductions have been hypothesized (Trinkaus, Churchill & Ruff, 1994; Sládek et al., 2016; Sparacello et al., 2017).

Extending these humeral robusticity trends deeper into the Pleistocene hominin record (e.g., *H. erectus*) has proven more challenging, among other reasons, due to the relative incompleteness of the fossil record. Based on initial work, humeral strength of African *H. erectus* (i.e., polar section modulus) appears to fit squarely within modern human levels of overall humeral strength Ruff (2008: Fig. 2). A similar quantitative assessment of Asian *H. erectus* humeral strength has not yet been performed, although levels of skeletal robusticity in more recent Late Pleistocene hominins from Asia have been carefully quantified and evaluated (Shackelford, 2007; Shang & Trinkaus, 2010; Sparacello et al., 2017). To date, evaluation of humeral strength in East Asian *H. erectus* still relies largely on the original descriptions of Zhoukoudian Humerus I and Humerus II published by Weidenreich (1938) and Weidenreich (1941), who remarked upon the slenderness of the Humerus II shaft along with comparably more prominent muscle markings on its external surface relative to modern human humeri. As with *H. erectus* femora from Zhoukoudian, Weidenreich (1938) and Weidenreich (1941) noted absolutely thicker cortical bone and narrower (circular) medullary canals in *H. erectus* humeri as evidence of stouter shafts compared to those of modern humans. Weidenreich (1941: 57) also portrayed differences in robusticity between Zhoukoudian and modern human humeral shafts as less than differences between their femoral shafts, even suggesting that Zhoukoudian *H. erectus* fell within the range of modern human variability in humeral robusticity.

Subsequent to the initial descriptions of Weidenreich (1941), a third partial hominin humerus (PA64, Humerus III) was recovered from Zhoukoudian Locality 1 and attributed to *H. erectus* (Woo & Chia, 1954). In assessing all three humeral fossils from Zhoukoudian, Antón (2003) made broad qualitative comparisons to approximately 1 Ma older African *H. erectus* humeri, namely those of KNM-ER 1808 and KNM-WT 15000. Antón (2003: 151) noted a narrower external breadth at the midshaft in Zhoukoudian humeri, presumably based on Humerus II and Humerus III, and that Humerus II was “equally long, and exhibits the typically thick cortical walls and reduced medullary cavity seen in African *H. erectus* fossils.” This characterization echoed the determination of Weidenreich (1941), in part, in suggesting that humeral structure of East Asian and African *H. erectus* differed from that of modern humans in similar ways (i.e., thicker cortical bone and narrower medullary cavities). What remains unknown, however, is whether a quantitative evaluation of humeral rigidity and strength in East Asian and African *H. erectus* can corroborate this suggested equivalence, and whether humeri from Zhoukoudian *H. erectus* may be truly modern human-like in their diaphyseal robusticity (i.e., relative humeral rigidity and strength).

The goals of the present study are threefold. First, we provide the first quantitative assessment of humeral rigidity and strength in East Asian *H. erectus*. Second, these new data will permit the first quantitative comparisons of humeral rigidity and strength in East Asian versus African *H. erectus*, which will contribute to an improved understanding of postcranial robusticity and variability within the taxon overall, much as recent investigations of *H. erectus* lower limb elements have (e.g., Puymerail et al., 2012; Ruff et al., 2015b). Specifically, we address whether East Asian and African *H. erectus* humeral diaphyses are similar in cortical thickness and medullary cavity dimensions by quantifying their cross-sectional geometry and strength properties. Comparisons between humeri of Zhoukoudian *H. erectus*, more recent Late Pleistocene Eurasian hominins, and two modern Chinese populations are also undertaken in order to better contextualize any potential uniqueness of Zhoukoudian humeral robusticity. Third, by including two modern Chinese populations that would be expected to exhibit similar latitudinal trends in ecomorphological body and limb proportions as earlier hominins from East Asia, we address whether East Asian *H. erectus* may exhibit the suggested modern human-like levels of humeral robusticity. In addition to providing new internal structural data for Zhoukoudian Humerus II and Humerus III, we provide a new detailed description of Humerus III surface morphology. This is intended to complement earlier descriptions of Humerus I and II by Weidenreich (1941), and to supplement an initial description of Humerus III by Woo & Chia (1954). Ultimately, the current study provides an opportunity to begin to place East Asian *H. erectus* humeral robusticity into broader temporal and regional hominin contexts.

## Material and Methods

The site of Zhoukoudian consists of a series of limestone caves approximately 50 km southwest of Beijing. It is situated in a transitional region between mountains and plains (Xie et al., 1985; Zhang, 2004). Excavations at Zhoukoudian Cave, Locality 1 were performed between 1921 and 1973. Dating Locality 1 has been attempted on several occasions using a variety of methods; adding the most recent cosmogenic efforts generates a potential estimated range of 0.68 Ma to 0.78 Ma (Shen et al., 2009). The Middle Pleistocene landscape of the immediate area was generally similar to the present landscape. Sporopollen and sediment analyses, as well as faunal composition, suggest that the surrounding area was mainly covered by forest and steppe, with each of these being alternately dominant over the course of the Zhoukoudian hominin occupation (Zhang & Tang, 2007). Hominins are thought to have occupied the cave itself, or lived near its opening in a rockshelter during the Middle Pleistocene, but the overall range of cave use is uncertain (Binford et al., 1985; Weiner et al., 1998; Wu, 1999).

**Figure 1:**
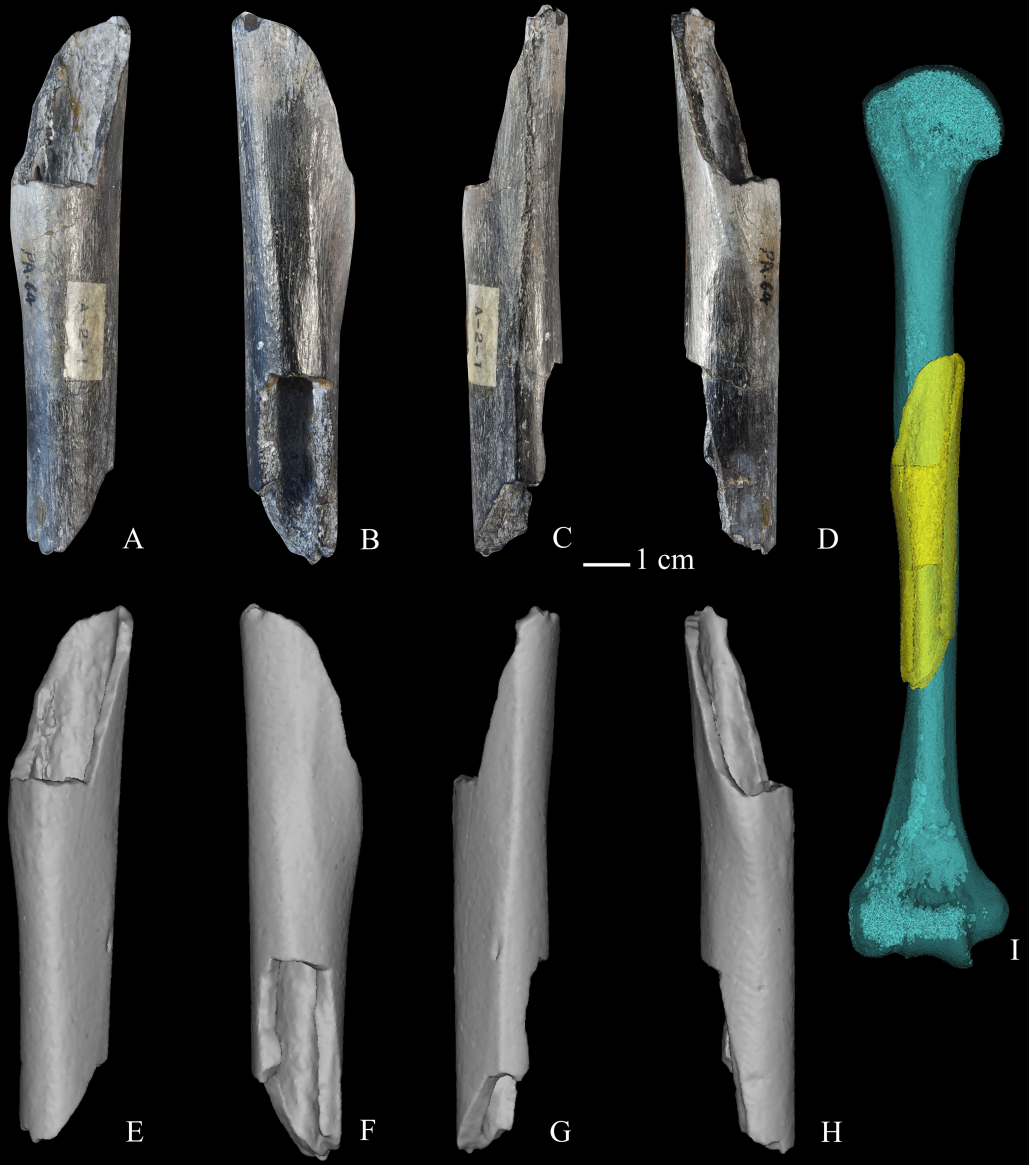
Zhoukoudian partial right humerus (PA64, Humerus III). (A) anterior view of the original fossil; (B) posterior view of the original fossil; (C) medial view of the original fossil; (D) lateral view of the original fossil; (E) anterior view of the virtual reconstruction; (F) posterior view of the virtual reconstruction; (G) medial view of the virtual reconstruction; (H) lateral view of the virtual reconstruction; (I) a rendering (yellow) created from Humerus III is superimposed on a mirrored rendering (light blue) created from the composite cast of Humerus II. Note general correspondence in external shape and morphology between the midshaft regions of Humerus II and Humerus III renderings. Weidenreich (1941) estimated maximum length of the Humerus II rendering as 324.0 mm.

A majority of original Zhoukoudian postcranial fossils disappeared in the 1940’s, and are represented today either by descriptions (e.g., Weidenreich, 1941; Weidenreich, 1943) or casts produced by Weidenreich. Weidenreich (1941) described two humeral specimens from Zhoukoudian Locality 1 (Humerus I and II), noting their general external rugosity compared to modern humans. Neither partial humerus was associated with other skeletal elements, although Weidenreich (1941: Table 1) raised the possibility that Humerus II could have been associated with femur 330 (Femur III). Weidenreich (1941) described Humerus I (specimen 81) as an unweathered small fragment of a left humerus, preserving a sharp lateral supracondylar ridge and adjoining parts of the anterolateral and posterior surfaces near the lateral margin of the olecranon fossa (see Weidenreich, 1941: Figs. 27–29). Based largely on the sharpness of its lateral supracondylar ridge, Weidenreich (1941) attributed Humerus I to a male individual. Weidenreich (1941) described Humerus II (specimen 319) as a substantial part of a left humeral diaphysis with irregular breaks through the shaft approximately 20–30 mm distal to its surgical neck and 55 mm proximal to its epicondyles (Weidenreich, 1941: Figs. 30–32). Weidenreich (1941) noted its robusticity and sharp surface contours, attributing it also to a male individual. Weidenreich (1941: Fig. 31) incorporated the more fragmentary Humerus I in his reconstruction of Humerus II, which he justified by pointing towards their similar external appearance and preserved proportions, arriving at a reconstructed maximum length of 324 mm for the composite left humerus. In 1951, a third partial hominin humerus (PA64, Humerus III) was discovered at Zhoukoudian Locality 1 and attributed to *H. erectus* (Woo & Chia, 1954). Humerus III is a right humeral fragment, preserving 108.2 mm (maximum dimension) of the middle region of the shaft (Fig. 1; see Text S1).

### Comparative samples

Zhoukoudian Humerus II and Humerus III were compared with African *H. erectus* (KNM-ER 1808), East Asian Late Pleistocene hominins, Middle Paleolithic modern humans, Neanderthals, European early Upper Paleolithic modern humans, and recent modern Chinese. Refer to Table S1 for individual specimens included in the comparative sample. Background information, such as associated dates and presumed general activity patterns of groups, are briefly summarized in Text S2 when available.

### Acquisition of cross-sectional properties

Humeri from Zhoukoudian *H. erectus*, the Late Pleistocene early modern human from Tianyuan Cave, and recent modern Chinese were scanned using the 450 kV high resolution computed tomography facilities (designed by the Institute of High Energy Physics, Chinese Academy of Sciences) housed in the Institute of Vertebrate Paleontology and Paleoanthropology (IVPP). Scan parameters for the sample included: 380 kV, 1.5 mA, 4 frame averaging, 0.5 angular increment, and 360 degrees of rotation. Final isometric voxel size obtained for the sample was 160 µm. For each scan, there were 720 projections converted into image stacks of .RAW files using the IVPP225kVCT_Recon algorithm.

**Figure 2:**
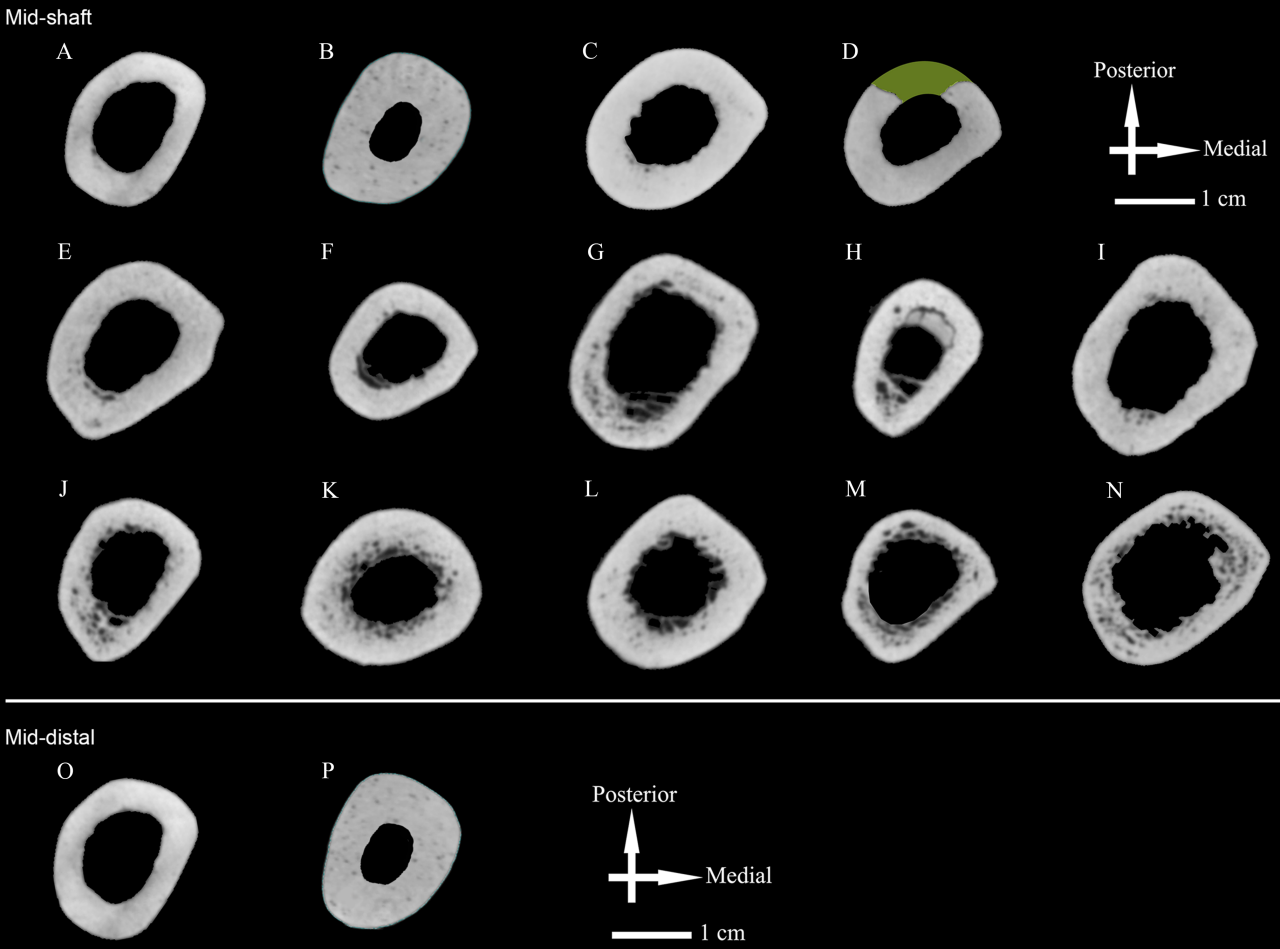
Humeral cross sections. (A) Zhoukoudian Humerus III; (B) Zhoukoudian Humerus II; (C) Tianyuan (right); (D) Tianyuan (left); (E) Datong-1; (F) Datong-2; (G) Datong-3; (H) Datong-4; (I) Datong-5; (J) Datong-6; (K) Datong-7; (L) Datong-8; (M) Datong-9; (N) Datong-10; (O) Zhoukoudian Humerus III; (P) Zhoukoudian Humerus II. In the upper three rows, midshaft cross sections are illustrated for Zhoukoudian Humerus II and Humerus III, Tianyuan 1 right and left humeri, and Datong humeri (*n* = 10). The reconstructed cross section from the left humerus of Tianyuan 1 has missing cortical bone estimated in green. In the bottom row, cross sections are illustrated for a second, more distal location of Zhoukoudian Humerus II and Humerus III. Both estimated cross sections from the Weidenreich composite cast of Humerus II have been mirrored for illustration purposes. All midshaft cross sections from the Junziqing humeri (*n* = 23) are illustrated in Fig. S1.

In order to quantify and compare internal structure, serial image data stacks obtained from high resolution scanning were imported into VGStudio Max 2.1 (Volume Graphics GmbH, Heidelberg, Germany). Using the region of interest tool, with a tolerance setting of 3,000, we selected all voxels representing the material of interest (i.e., a fossil or modern comparative humerus). From the selected voxels, a 3D volume or region was created, and from each of these a volume rendering of an entire bone was extracted. Each volume rendering of a comparative specimen was aligned to the same vertical and horizontal axes *in silico* as have been used for physical specimens. In other words, criteria for aligning humeral volume renderings followed standard procedures used with dry bones (Ruff, 2002a; Carlson, 2005), and that have been adapted for use in *in silico* environments (Carlson et al., 2008). Briefly, the longitudinal axis of a rendered diaphysis was aligned to a vertical axis in morphospace. Next, each rendered volume was aligned to a vertical plane passing through this vertical axis by rotating the 3D rendering about its longitudinal (now also vertical) axis, or about its midpoint (i.e., rotating end over end), until the two most anterior points of the distal epiphysis (i.e., usually on the capitulum and trochlea of the rendering, or on both rims of the trochlea of the rendering) and the most anterior projecting point on the proximal end (e.g., usually the lesser tubercle) were positioned in the same vertical plane. Once specimens were aligned, intact diaphyseal cross sections were obtained from the midshaft of the rendering and saved as 16-bit TIF images (Fig. 2 and Fig. S1). Additional details on the alignment of diaphyses and derivation of cross sections from Humerus II and Humerus III are reported in the Supplementary Information (see Text S3).

Once cross sections were acquired (Fig. 2 and Fig. S1), they were imported into ImageJ 1.50e (Rasband, 2015) where they were converted to 8-bit TIFF images and standard cross-sectional properties were calculated using the BoneJ 1.4.1 plugin (Doube et al., 2010). The only property not measured using the BoneJ 1.4.1 plugin (Doube et al., 2010) was total subperiosteal area (TA), which we measured using the magic wand tool in ImageJ 1.50e (Rasband, 2015). In order to pre-process the 8-bit TIFFs for use in BoneJ, a three-step process was followed. First, each image was binarized using a threshold for inclusion equal to the half-maximum gray value amongst bone pixels. Second, the endosteal border of each cross section was cleaned (e.g., trabecular struts digitally removed) following criteria outlined elsewhere (Carlson, 2005). Third, internal spaces between endosteal and periosteal envelopes were filled, thus creating a cross section without intracortical porosity.

For descriptive and comparative purposes, we report TA, cortical area (CA), percentage cortical area (%CA), and principal moments of area (*I*_max_ and *I*_min_). We calculate polar moment of area (*J*) as the sum of *I*_max_ and *I*_min_. We also report section moduli (*Z*_max_ and *Z*_min_) and the polar section modulus (*Z*_p_). We select these properties, which are calculated independent of anatomical axes, in recognition of the possibility that the fully reconstructed articular ends of the composite cast of Humerus II may introduce an unknown amount of error when trying to precisely identify anteroposterior (AP) and mediolateral (ML) anatomical planes during the alignment procedure described above. Thus, we did not calculate any structural properties with respect to AP or ML anatomical planes (i.e., *I*_x_, *I*_y_, *Z*_x_, and *Z*_y_) for either Humerus II or Humerus III.

### Standardization and analysis of structural properties

When comparing diaphyseal cross-sectional properties of long bones across disparate groups sampling different latitudes, particularly within the lower limb, it is important to standardize properties by measures of body size or shape because the former may exhibit allometric relationships with the latter (Ruff et al., 1993; Ruff & Larsen, 2014). Such standardized properties are reliable and accurate measures of skeletal robusticity (see Pearson, 2000). Typically, body mass is the most frequently used proxy for body size (or force applied when modelling beam bending), while bone length is the most frequently used proxy for beam length. Thus, a measure such as the product of body mass and bone length is appropriate for scaling second moments of area or the polar moment of area (Polk et al., 2000), and section moduli (Ruff, 2003) by approximating bending moments of long bones.

For specific interregional comparisons, such as those of East Asian and African *H. erectus* properties, we followed the aforementioned rationale and standardized second moments of area, polar moments of area, and section moduli using the product of estimated body mass and bone length to account for any potential ecomorphological trends in body proportions. For Humerus II and Humerus III, we derived body mass estimates emphasizing the average (53.6 kg) within a range of ± one standard deviation (1.7 kg) calculated from multivariate body mass estimates for Femur I (54.8 kg), Femur IV (54.3 kg), and Femur VI (51.6 kg) (Grabowski et al., 2015). Weidenreich (1941) attributed Femur I, Femur IV, and Femur VI to male individuals, as he also attributed the reconstructed composite cast of Humerus II. For KNM-ER 1808, we derived an estimated body mass emphasizing the average (60.2 kg) within a range of ±one standard deviation (20.4 kg) calculated from three recently published estimates: 79 kg (Will & Stock, 2015), 63 kg (Antón, Potts & Aiello, 2014: Table S2), and 38.5 kg (Grabowski et al., 2015). The comparatively lower estimate reported by Grabowski et al. (2015) may be influenced by their use of cadaveric specimens, which have been shown to lead to equations that underestimate body mass (Ruff et al., in press). Shang & Trinkaus (2010) used vertical femoral head diameter and several regression formulae to calculate a range of body mass estimates for Tianyuan 1. Ultimately, they endorsed a body mass estimate of 85.1 kg for scaling limb bone structural properties of Tianyuan 1, which is the value we adopted in the present study. For Middle Paleolithic, Neanderthal, Early Upper Paleolithic, and Late Upper Paleolithic hominins, we used body mass estimates reported by Sparacello et al. (2017).

Based on reasonably similar external dimensions and contours in their overlapping regions (see Figs. 1 and 2), we used estimated length of the composite Humerus II reconstruction as a suitable proxy for estimated length of Humerus III. However, in acknowledgement of the uncertainty that exists in estimating the length of Humerus II, and by default Humerus III, we generated three different length estimates for standardizing both sets of cross-sectional properties. For the first estimate, we used maximum length (324.0 mm) of the composite Humerus II reconstruction published by Weidenreich (1941) (Figs. S2 and S3). Weidenreich (1941: 55) remarked that the proximal end of the reconstruction “may possibly have been shorter than appears in the restoration.” For this reason, the estimate of Weidenreich serves as a reasonable upper boundary for our range of length estimates. For the second estimate, since the composite Humerus II reconstruction retained the deltoid tuberosity and the proximal border of the olecranon fossa, we regressed distance between the distal-most extent of the deltoid tuberosity and the proximal-most extent of the olecranon fossa against maximum length in the recent modern Chinese sample (*n* = 33; Maximum length = (distance between distal margin of deltoid tuberosity and proximal margin of olecranon fossa) (1.544) + (133.172); *p* < 0.001; R-squared = 0.551; see Text S4 for more details; Table S2, and Figs. S2–S4). The regression-derived estimate of Humerus II maximum length is 307.4 mm. Since both modern Chinese groups, particularly the Junziqing, tended to have shorter humeri than other groups in the sample, and notably overlapped with the upper half of the published range for the East Eurasian Late Upper Paleolithic sample (Table 1), this estimate serves as a reasonable lower boundary for our range of length estimates. Finally, we averaged both of these estimates to derive a third maximum length (315.7 mm). All three estimates were utilized separately when standardizing cross-sectional properties, creating a range of length values (16.6 mm) equal to approximately 5.3% of the average length estimate (315.7 mm). For KNM-ER 1808, we used a rough approximation of 350 mm for its estimated length (Ruff, 2008; C Ruff, pers. comm., 2016). For Tianyuan 1, we used a biomechanical length of the left humerus (327.4 mm), as reported by Shang & Trinkaus (2010). We used the same value (327.4 mm) as a proxy for length of the right humerus of Tianyuan 1, which has not yet been estimated. For Middle Paleolithic, Neanderthal, Early Upper Paleolithic, and Late Upper Paleolithic hominins, we used humeral lengths reported by Sparacello et al. (2017). For Datong and Junziqing recent modern human samples, we measured and reported humeral maximum length.

**Table 1:** Midshaft humeral unstandardized properties of Zhoukoudian right humerus (III) and comparative samples.

		Length	Body Mass	TA	CA	%CA	*I*_max_	*I*_min_	*Z*_max_	*Z*_min_	*J*	*Z*_p_
		(mm)	(kg)	(mm^2^)	(mm^2^)		(mm^4^)	(mm^4^)	(mm^3^)	(mm^3^)	(mm^4^)	(mm^3^)
Zhoukoudian III^a^^,^^b^		307.4 −324.0	53.6 ± 1.7	250	167	66.8	5,959	3,307	579	415	9,266	875
KNM-ER 1,808^c^		350.0	60.2 ± 20.4	240	197	82.1	5,212	3,891	503	457	9,103	877
Tianyuan 1^a^^,^^d^		327.4	85.1	330	249	75.5	10,561	6,345	912	684	16,906	1,391
Middle Paleolithic Modern Human^e^ (*n* = 4 for length, TA, CA, *I*_x_, and *I*_y_, *n* = 2 for body mass, *n* = 5 for *I*_max_, *I*_min_, and *J*)	Mean	358.3	66.1	303.5	235.3	76.2	8,152	5,216	–	–	13,368	–
	S.D.	20.5	3.9	80.5	81.3	7.4	4,452	2,985	–	–	7,395	–
	Min	329.0	63.3	190.7	130.0	68.2	3,591	1,946	–	–	5,537	–
	Max	375.0	68.8	381.4	327.4	85.8	14,567	8,834	–	–	23,401	–
Neanderthal^e^ (*n* = 12 for length, *n* = 9 for body mass, *n* = 12 for TA, CA, *I*_x_, and *I*_y_, *n* = 14 for *I*_max_ and *I*_min_, *n* = 15 for *J*)	Mean	301.6	71.5	314.8	244.5	77.8	9,373	5,444	–	-	14,945	–
	S.D.	20.6	10.1	79.3	65.6	7.7	4,062	2,479	–	–	6,246	–
	Min	262.0	59.9	183.3	125.3	61.8	3,705	1,887	–	–	5,592	–
	Max	335.5	85.5	426.0	365.9	88.1	14,787	9,757	–	–	24,544	–
Early Upper Paleolithic Modern Human^e^ (*n* = 17 for length, *n* = 13 for body mass, *n* = 14 for TA, CA, *I*_x_, and *I*_y_, *n* = 22 for *I*_max_, *I*_min_, and *J*)	Mean	332.6	69.0	330.7	227.4	69.6	9,317	6,094	–	–	15,411	–
	S.D.	25.9	7.8	73.4	48.6	9.2	3,558	2,253	–	–	5,716	–
	Min	284.0	55.7	181.5	143.0	52.4	3,210	2,207	–	–	5,417	–
	Max	371.0	82.5	444.2	316.8	91.1	17,592	10,579	–	–	27,736	–
East Eurasia Late Upper Paleolithic Modern Human^e^ (*n* = 9 for length, *n* = 8 for body mass, *n* = 10 for TA, CA, *I*_x_, *I*_y_, *I*_max_, *I*_min_, and *J*)	Mean	274.3	51.4	232.1	172.5	74.7	5,612	2,937	–	–	8,549	
	S.D.	18.1	9.9	30.5	18.7	5.1	1,570	774	–	–	2,251	
	Min	252.0	42.3	189.5	153.6	66.5	3,671	2,132	–	–	5,803	
	Max	311.0	70.5	283.1	218.0	84.6	8,331	4,486	–	–	12,817	
Datong (*n* = 10)^f^	Mean	305.8	–	308	193	62.8	8,660	5,360	742	548	14,020	1,143
	S.D.	18.2	–	69	46	5.7	3,743	2,254	251	196	5,951	395
	Min	272.4	–	210	131	54.4	4,134	2,166	401	307	6,336	601
	Max	328.0	–	397	258	69.0	14,107	8,751	1,072	831	22,858	1,715
Junziqing (*n* = 23)^f^	Mean	286.2	–	268	161	59.7	6,199	3,958	565	451	10,157	915
	S.D.	17.5	–	50	44	10.8	2,514	1,663	190	143	4,132	308
	Min	262.9	–	193	90	42.9	2,678	1,722	288	255	4,632	497
	Max	327.7	–	384	243	78.8	11,814	7,540	988	738	18,877	1,571

**Notes.**

a Estimated cross section location due to incomplete length.

b Maximum length of the left Zhoukoudian Humerus II was reported by Weidenreich (1941) to be 324.0 mm. We estimated maximum length as 307.4 mm using a regression analysis of the distance between the deltoid tuberosity and the proximal margin of the olecranon fossa against maximum length on our comparative sample of Datong and Junziqing modern *Homo sapiens* (*n* = 33; see Text S4). In order to be conservative, we use both estimates to provide a range of standardized values for Zhoukoudian humeri about a mean value (315.7 mm). In order to standardize cross-sectional properties, we used maximum length estimates of the reconstructed left Zhoukoudian Humerus II as proxies for maximum length estimates of the partial right Zhoukoudian Humerus III.

c Cross-sectional data for a 40% length section published by Ruff (2008: Fig. 1). We used a rough approximation of 350.0 mm for humeral length (Ruff, 2008; C Ruff, pers. comm., 2016).

d In order to standardize cross-sectional properties, but acknowledging substantial bilateral asymmetry in their cross-sectional properties, we chose to use biomechanical length of the left Tianyuan 1 humerus (327.4 mm: Shang & Trinkaus, 2010) as a proxy for length of the right Tianyuan 1 humerus.

e Data from Churchill (1994), Trinkaus, Churchill & Ruff (1994), Trinkaus & Churchill (1999), Crevecoeur (2008), and Sparacello et al. (2017).

f Amongst the recent modern human comparative sample, the distal-most point of the deltoid tuberosity was between 43 and 53% shaft length, with the majority of specimens falling between 46 and 51%.

While some have argued that similar scaling factors should apply to the upper limb as well as the lower limb, as correlations between humeral properties and body mass have been demonstrated (Ruff, 2000; Ruff, 2003), others have argued on theoretical grounds that in humans upper limb loading should be less influenced by body mass than lower limb loading since the upper limbs are not habitually weight-bearing (Pearson, 2000; Carlson, Grine & Pearson, 2007). In the present study, since the humeral diaphysis is less likely affected by potential body breadth differences compared to the proximal femur, and since many individuals within our region-specific East Asian sample were without reliable body mass estimates (e.g., no associated femoral head measurements), we follow others who used only bone length to standardize diaphyseal properties (Trinkaus & Churchill, 1999), particularly for the humerus (Trinkaus et al., 1999; Pearson, 2000; Carlson, Grine & Pearson, 2007). We emphasize this additional standardization protocol when conducting intraregional comparisons between Zhoukoudian *H. erectus*, Tianyuan 1, and the recent modern Chinese samples, for whom ecomorphological trends in body or limb proportions are expected to be relatively consistent. For such comparisons, we standardize cross-sectional properties to create dimensionless values as follows: total area and cortical area were divided by the square of maximum length, section moduli were divided by the third power of maximum length, and humeral principal or polar moments of area were divided by the fourth power of maximum length.

## Results

### Are East Asian and African *H. erectus* humeral diaphyses similar in cortical thickness and medullary cavity dimensions?

The midshaft of Humerus II exhibits a relatively high estimate of %CA similar to the %CA of the KNM-ER 1808 cross section, both being near the upper end of the observed hominin ranges (Tables 1 and 2). The more distal cross section of Humerus II exhibits a similar trend (i.e., 2.8% lower %CA than its midshaft), still exceeding the %CA of the KNM-ER 1808 cross section (Table 2 and Table S3). The midshaft of Humerus III, on the other hand, is comparatively lower in %CA, falling usually in the lower half of the observed hominin group ranges (i.e., between observed group means and minimum values) (Table 1). While the more distal cross section of Humerus III, like Humerus II, also exhibits an incremental difference in %CA compared to its midshaft (0.4% lower: Table 1 and Table S3), it still usually falls in the lower half of the observed hominin group ranges. Due to the similarity in %CA between the two locations, only the midshaft of Humerus II and Humerus III is considered further.

**Table 2:** Midshaft humeral unstandardized properties of Zhoukoudian left humerus (II) and comparative samples.

		Length	Body mass	TA	CA	%CA	*I*_max_	*I*_min_	*Z*_max_	*Z*_min_	*J*	*Z*_p_
		(mm)	(kg)	(mm^2^)	(mm^2^)		(mm^4^)	(mm^4^)	(mm^3^)	(mm^3^)	(mm^4^)	(mm^3^)
Zhoukoudian II^a^^,^^b^		307.4–324.0	53.6 ± 1.7	261	228	87.4	6,985	4,143	640	518	11,128	1,009
Tianyuan 1^a^^,^^c^		327.4	85.1	252	190	75.4	5,931	3,868	603	463	9,799	928
Middle Paleolithic Modern Human^d^ (*n* = 2 for length, body mass, TA, CA, *I*_x_, and *I*_y_, *n* = 3 for *I*_max_, *I*_min_, and *J*)	Mean	353.3	68.9	283.1	217.0	76.8	5,894	4,088	–	–	9,981	–
	S.D.	30.8	0.1	5.2	56.9	21.5	2,021	1,619	–	–	3,618	–
	Min	331.5	68.8	279.4	176.7	61.6	3564	2,287	–	–	5,851	–
	Max	375.0	69.0	286.7	257.2	92.1	7,170	5,421	–	–	12,591	–
Neanderthal^d^ (*n* = 5 for length, *n* = 4 for body mass, *n* = 7 for TA and CA, *n* = 6 for *I*_x_ and *I*_y_, *n* = 8 for *I*_max_ and *I*_min_, *n* = 9 for *J*)	Mean	314.4	79.1	256.0	197.8	77.6	7,879	4,173	–	–	12,112	–
	S.D.	13.4	9.7	44.0	29.3	3.6	2,863	1,658	–	–	4,199	–
	Min	299	64.8	203.5	170.7	73.9	4,629	2,250	–	–	6,879	–
	Max	334	85.5	341.1	251.9	84.2	12,020	6,411	–	–	18,250	–
Early Upper Paleolithic Modern Human^d^ (*n* = 20 for length, *n* = 15 for body mass, *n* = 17 for TA, CA, *I*_x_, and *I*_y_, *n* = 22 for *I*_max_ and *I*_min_, *n* = 23 for *J*)	Mean	326.5	68.4	298.6	198.6	67.1	7,119	4,799	–	–	12,138	–
	S.D.	21.0	7.7	46.1	29.5	8.9	1,965	1,315	–	–	2,978	–
	Min	288.0	54.3	199.8	133.0	47.9	3,670	2,148	–	–	5,895	–
	Max	370.0	82.5	394.1	246.7	83.0	10,701	7,316	–	–	17,605	–
East Eurasia Late Upper Paleolithic Modern Human^d^ (*n* = 7 for length, *n* = 5 for body mass, *n* = 10 for TA, CA, *I*_x_, *I*_y_, *I*_max_, *I*_min_, and *J*)	Mean	273.1	53.2	227.6	168.4	74.2	5,106	2,972	–	–	8,078	–
	S.D.	20.3	10.5	33.8	27.9	7.6	1463	955	–	–	2,395	–
	Min	250.0	42.3	186.7	138.8	65.7	3,437	1,900	–	–	5,587	–
	Max	311.0	70.5	281.8	225.1	86.5	7,432	4,724	–	–	11,968	–

**Notes.**

a Estimated cross section location due to incomplete length.

b Maximum length of the left Zhoukoudian Humerus II was reported by Weidenreich (1941) to be 324.0 mm. We estimated maximum length as 307.4 mm using a regression analysis of the distance between the deltoid tuberosity and the proximal margin of the olecranon fossa against maximum length on our comparative sample of Datong and Junziqing modern *Homo sapiens* (*n* = 33; see Text S4). In order to be conservative, we use both estimates to provide a range of standardized values for Zhoukoudian humeri about a mean value (315.7 mm). We estimated cross-sectional properties of Humerus II from its periosteal contour, and a radiograph published by Weidenreich (1941: Fig. 58B); see Text S3.

c Data from Shang & Trinkaus, 2010.

d Data from Churchill (1994), Trinkaus, Churchill & Ruff (1994), Trinkaus & Churchill (1999), Crevecoeur (2008), and Sparacello et al. (2017).

**Figure 3:**
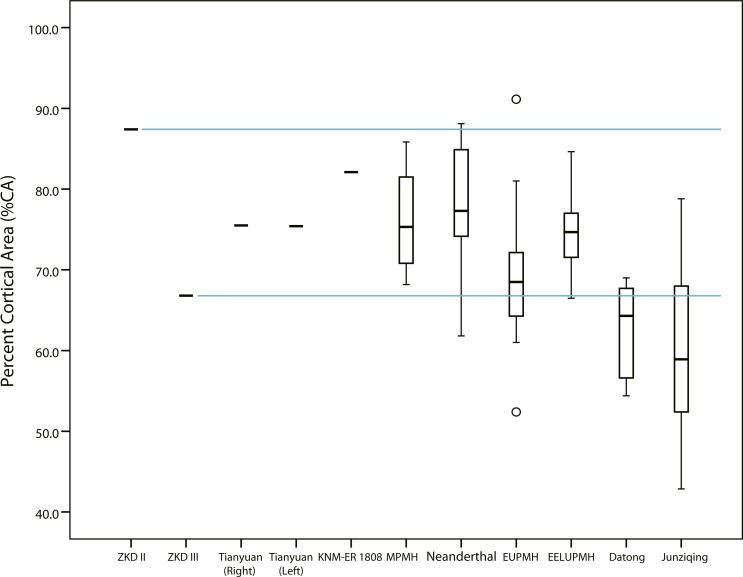
Box plots of percent cortical area (%CA) in humeral midshaft cross sections reported in Tables 1 and 2. Solid horizontal lines within boxes indicate median values, while height of boxes indicates interquartile range (i.e., contains 50% of observations) and whiskers indicate the observed highest and lowest values that do not exceed 1.5 times the interquartile range. Note that the cross section for KNM-ER 1808 is an estimated 40% diaphyseal length rather than midshaft (Ruff, 2008). ZKD, Zhoukoudian; MPMH, Middle Paleolithic Modern Human; EUPMH, Early Upper Paleolithic Modern Human; EELUPMH, East Eurasia Late Upper Paleolithic Modern Human.

Midshaft %CAs of both Tianyuan 1 humeri fall approximately midway between the observed lower Humerus III midshaft %CA and the estimated higher Humerus II midshaft %CA, as do average %CAs for the Middle Paleolithic, Neanderthal, and East Eurasian Late Upper Paleolithic groups (Tables 1 and 2, Fig. 3). Average %CA of the Early Upper Paleolithic group also exceeds the observed %CA of the Humerus III midshaft, although by only roughly half the amount of the other Late Pleistocene hominin groups. Cognizant of the generally equivalent subperiosteal areas in midshaft cross sections of Humerus II and Humerus III versus the cross section of the KNM-ER 1808 humerus (i.e., observed differences are less than 5%), thicker cortical bone and a relatively reduced medullary cavity best characterize Humerus II and the KNM-ER 1808 humerus rather than Humerus III.

When standardizing the amount of bone in midshaft cross sections by squared humeral length (sCA: Table S4), the range of observed Humerus III values tends to fall above sCA of KNM-ER 1808. The same trend is evident when substituting sCA of the more distal cross section of Humerus III (Table S3). By comparison, ranges of estimated Humerus II sCAs from the midshaft (Table S5) and the more distal cross section (Table S3) fall well above those of either of the other *H. erectus* humeri (Tables S3–S5). A comparison of CAs standardized to average body mass estimates largely supports the same trend where Humerus II (4.25) exceeds the values exhibited by other *H. erectus* humeri: Humerus III (3.12) and KNM-ER 1808 (3.27) (Tables 1 and 2). With few exceptions, and irrespective of the estimated lengths used as scaling factors in the present study, estimated sCAs of the Humerus II midshaft fit comfortably within the upper half of observed sCA ranges for left humeri of Late Pleistocene hominins (i.e., between observed group means and maximum values) (Table S5), while observed sCAs of the Humerus III midshaft tend to fall within the lower half of the observed sCA ranges for right humeri of Late Pleistocene hominins (i.e., between observed group means and minimum values) (Table S4). The observed sCA for the KNM-ER 1808 cross section, on the other hand, falls below the observed midshaft values of both right and left Tianyuan I humeri, as well as in the lower half of the observed sCA ranges for right humeral midshafts of all other hominin groups in the study. In other words, despite the comparatively high %CA demonstrated by KNM-ER 1808 (i.e., its relatively high cortical thickness), its rather long estimated length of 350 mm (Ruff, 2008), which falls in the upper end of the range of humeral lengths for the entire comparative sample analyzed in the present study, results in relatively lower amounts of length-standardized compressive rigidity compared to the Zhoukoudian humeri.

**Table 3:** Midshaft humeral standardized properties (by estimated body mass × maximum length) of Zhoukoudian right humerus (III) and comparative samples.^*^

	BM	HL	*sI*_max_	*sI*_min_	*sZ*_max_	*sZ*_min_	*sJ*	*sZ*_p_
ZKD Humerus III	53.6	307.4	0.362	0.201	0.035	0.025	0.562	0.053
	**53.6**	**315.7**	**0.352**	**0.195**	**0.034**	**0.0245**	**0.548**	**0.052**
	53.6	324.0	0.343	0.190	0.033	0.024	0.534	0.050
	55.3	307.4	0.351	0.195	0.035	0.025	0.545	0.051
	55.3	315.7	0.341	0.189	0.034	0.025	0.531	0.050
	55.3	324.0	0.333	0.185	0.033	0.024	0.517	0.049
	51.9	307.4	0.374	0.207	0.034	0.024	0.581	0.055
	51.9	315.7	0.364	0.202	0.033	0.024	0.567	0.053
	51.9	324.0	0.354	0.197	0.032	0.023	0.551	0.052
KNM-ER 1,808	**60.2**	**350**	**0.247**	**0.185**	**0.024**	**0.022**	**0.432**	**0.042**
	80.6	350	0.185	0.138	0.018	0.016	0.323	0.031
	39.8	350	0.374	0.279	0.036	0.033	0.653	0.063
Tianyuan 1	85.1	327.4	0.379	0.228	0.033	0.025	0.607	0.050
Middle Paleolithic Modern Human (*n* = 2)		Mean	0.339	0.282	–	–	0.682	–
		S.D.	0.099	0.047	–	–	0.146	–
		Min	0.329	0.249	–	–	0.579	–
		Max	0.469	0.315	–	–	0.785	–
Neanderthal (*n* = 8 for sTA, sCA, sImax, and sImin, *n* = 9 for *sJ*)		Mean	0.420	0.244	–	–	0.682	–
		S.D.	0.165	0.117	–	–	0.265	–
		Min	0.222	0.100	–	–	0.322	–
		Max	0.668	0.441	–	–	1.109	–
Early Upper Paleolithic Modern Human (*n* = 7 for sTA and sCA, *n* = 13 for sImax, sImin, and *sJ*)		Mean	0.402	0.266			0.668	–
		S.D.	0.094	0.062			0.152	–
		Min	0.283	0.195			0.478	–
		Max	0.587	0.400			0.926	–
East Eurasian Late Upper Paleolithic Modern Human (*n* = 7)		Mean	0.414	0.217			0.631	–
		S.D.	0.109	0.030			0.132	–
		Min	0.321	0.175			0.509	–
		Max	0.636	0.259			0.875	–

**Notes.**

* Humeral lengths (HL), body masses (BM), and original properties used in calculating the standardized properties are reported in Table 1, except for ZKD humeri, where three length estimates (307.4, 315.7, and 324.0 mm) and three body mass estimates (Average + 1SD = 55.3 kg, Average = 53.6 kg, Average − 1SD = 51.9 kg) were used. Three body mass estimates of KNM-ER 1808 (Average + 1SD = 80.6 kg, Average = 60.2 kg, Average − 1SD = 39.8 kg) were also used. Bold font indicates values standardized by average length and body mass estimates.

### Are East Asian and African *H. erectus* humeral diaphyses similar in relative rigidity and strength?

Despite relatively small differences between subperiosteal areas (TA) of Zhoukoudian Humerus II and Humerus III midshafts (<3%: Tables 1 and 2), the observed differences in cortical thickness create about 15% greater unstandardized principal moments of area (*I*_max_ and *I*_min_) and polar moments of area (*J*) in Humerus II (Tables 1 and 2). The latter structural differences dissipate in the more distal cross section (<3%), being offset by a relative increase in subperiosteal area of Humerus III (Fig. 2; Table S3). This variability is noteworthy when comparing all *H. erectus* humeri. Humerus III, despite exhibiting markedly less cortical thickness than the humerus of KNM-ER 1808, still exhibits higher absolute *I*_max_, *J*, and *Z*_max_ than KNM-ER 1808 (Table 1 and Table S3). This indicates that Humerus III, despite its lower cortical thickness, retains comparatively more absolute rigidity or strength than the humerus of KNM-ER 1808 largely because of its relative expansion in external (subperiosteal) contour. Humerus II, by comparison, exhibits comparatively greater absolute rigidity or strength both because of its cortical thickness and its expanded external (subperiosteal) contour.

Standardizing structural properties results in different trends. When standardizing humeral rigidity or strength to the product of body mass and bone length, relative robusticity of Zhoukoudian humeri becomes even more apparent (Tables 3 and 4). Even the less thick of the two Zhoukoudian humeri (Humerus III), whether for the midshaft or the more distal cross section, consistently exceeds KNM-ER 1808 in each quantitative measure irrespective of the estimated length that is combined with the average estimate of body mass (Table 3 and S4). Only if the minimum estimate of body mass is used for standardizing properties of KNM-ER 1808 does Humerus III consistently fall below it, but Humerus II still slightly exceeds KNM-ER 1808 in some properties (e.g., *sI*_max_ and *sZ*_max_) and falls slightly below it in others (e.g., *sI*_min_, *sZ*_min_, and *sZ*_p_). Notably, KNM-ER 1808 falls near or below the means of comparably standardized structural properties of Late Pleistocene right humeri included in the study, even when using the minimum estimate of body mass (Table 3).

**Table 4:** Midshaft humeral standardized properties (by estimated body mass × maximum length) of Zhoukoudian left humerus (II) and comparative samples.^*^

	BM	HL	*sI*_max_	*sI*_min_	*sZ*_max_	*sZ*_min_	*sJ*	*sZ*_p_
ZKD Humerus II	53.6	307.4	0.424	0.251	0.039	0.031	0.675	0.061
	**53.6**	**315.7**	**0.413**	**0.245**	**0.038**	**0.0306**	**0.658**	**0.060**
	53.6	324.0	0.402	0.239	0.037	0.030	0.641	0.058
	55.3	307.4	0.411	0.244	0.038	0.030	0.655	0.059
	55.3	315.7	0.400	0.237	0.037	0.030	0.637	0.058
	55.3	324.0	0.390	0.231	0.036	0.029	0.621	0.056
	51.9	307.4	0.438	0.260	0.040	0.032	0.698	0.063
	51.9	315.7	0.426	0.253	0.039	0.032	0.679	0.062
	51.9	324.0	0.415	0.246	0.038	0.031	0.662	0.060
Tianyuan 1	85.1	327.4	0.213	0.139	0.022	0.017	0.352	0.033
Middle Paleolithic Modern Human (*n* = 2)		Mean	0.291	0.207			0.498	
		S.D.	0.031	0.043			0.074	
		Min	0.269	0.177			0.446	
		Max	0.313	0.237			0.550	
Neanderthal (*n* = 4 for sTA, sCA, and *sJ*, *n* = 3 for *sI*_max_ and *sI*_min_)		Mean	0.363	0.182			0.534	
		S.D.	0.186	0.102			0.237	
		Min	0.253	0.118			0.375	
		Max	0.578	0.300			0.877	
Early Upper Paleolithic Modern Human (*n* = 9 for sTA and sCA, *n* = 14 for *sI*_max_ and *syI*_min_, *n* = 15 for *sJ*)		Mean	0.300	0.201			0.506	
		S.D.	0.059	0.040			0.092	
		Min	0.202	0.129			0.355	
		Max	0.405	0.272			0.674	
East Eurasian Late Upper Paleolithic Modern Human (*n* = 5)		Mean	0.313	0.186			0.500	
		S.D.	0.044	0.035			0.077	
		Min	0.256	0.139			0.416	
		Max	0.353	0.215			0.566	

**Notes.**

* Humeral lengths (HL), body mass (BM), and original properties used in calculating the standardized properties are reported in Table 2, except for ZKD humeri, where three length estimates (307.4, 315.7, and 324.0 mm) and three body mass estimates (Average + 1SD = 55.3 kg, Average = 53.6 kg, Average − 1SD = 51.9 kg) were used. Bold font indicates values standardized by average length and body mass estimates.

**Figure 4:**
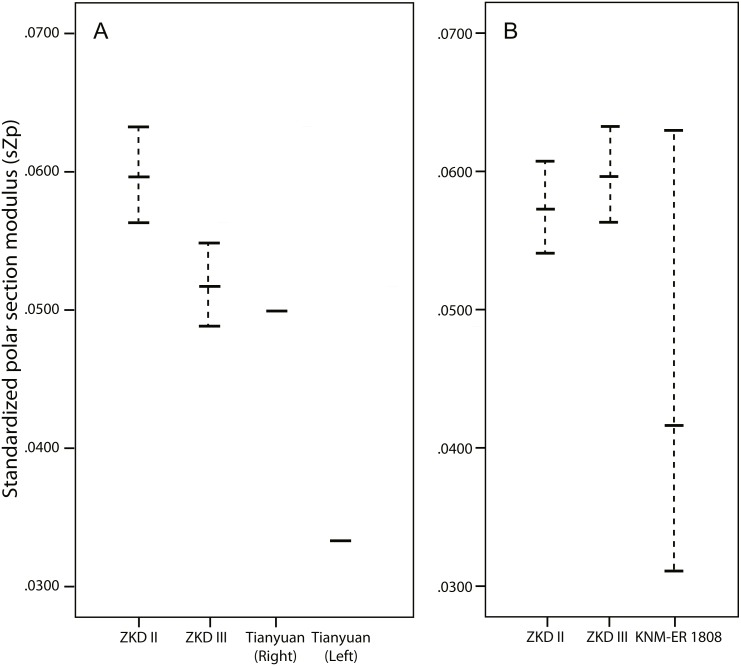
Line plots of standardized polar section modulus (*Z*_*p*_). Line plots of standardized polar section modulus (*Z*_*p*_) from the humeral midshaft (A) and mid-distal (B) diaphysis reported in Tables 3–4 and Table S3, respectively. Standardization procedures are reported in the methods section. The dotted lines illustrated for Zhoukoudian and KNM-ER 1808 indicate the range of standardized properties using different combinations of humeral length and body mass. The solid horizontal line within the range indicates the value of sZp standardized by average humeral length*average body mass. ZKD, Zhoukoudian.

The upper end of the range of Humerus III midshaft values consistently falls at or just below the *sI*_max_, *sZ*_max_, *sJ*, or *sZ*_p_ of the right Tianyuan 1 humerus (Table 3, Fig. 4, and Fig. S5), while the same Humerus III ranges consistently exceed those of the less strong left Tianyuan I humerus (Table 4, Fig. 4, and Fig. S5). By comparison, ranges of *sI*_max_, *sZ*_max_, *sJ*, and *sZ*_p_ estimated from the Humerus II midshaft consistently exceed those observed in either Tianyuan 1 humerus (Tables 3 and 4, Fig. 4, and Fig. S5). Compared to right humeri from other Late Pleistocene hominins (Table 3 and Fig. S5), the midshaft of Humerus III exhibits ranges of *sI*_max_, *sI*_min_, and sJ that usually overlap with the lower half of observed ranges (Neanderthals, Early Upper Paleolithic modern humans, East Eurasian Late Upper Paleolithic modern humans), or falls below them (Middle Paleolithic group; except for *sI*_max_). Compared to left humeri from other Late Pleistocene hominins (Table 4 and Fig. S5), the midshaft of Humerus II exhibits ranges of *sI*_max_, *sI*_min_, and *sJ* that overlap with the upper half of observed ranges (Neanderthals and Early Upper Paleolithic modern humans), or usually falls above them (Middle Paleolithic groups and East Eurasian Late Upper Paleolithic groups).

### Does East Asian *H. erectus* exhibit modern human-like humeral robusticity compared to two recent modern Chinese populations?

Weidenreich (1941) described Zhoukoudian humeri as modern-like in their robusticity. When comparing sCA of recent modern Chinese right humeri and Zhoukoudian humeri, the less robust right Humerus III overlaps within the bottom half of sCA ranges of both groups (Table S4), while the more robust left Humerus II overlaps with the upper half of sCA ranges of both groups (Tables S4 and S5). This overlap appears to be more attributable to the comparatively thick cortical shafts of both Zhoukoudian humeri rather than any sort of subperiosteal expansion since even the less robust Humerus III has a %CA that falls in the upper end of the ranges observed in both recent modern Chinese samples (Tables 1 and 2).

When comparing length-standardized humeral midshaft properties used to evaluate rigidity or strength, Humerus II usually overlaps with the lower half of the observed Datong ranges (i.e., between the observed group mean and minimum value) or falls below it, and overlaps entirely with the observed lower half of the less robust Junziqing ranges (Tables S4 and S5). Comparing length-standardized humeral properties of the right Humerus III to the equivalent properties of the recent modern Chinese right humeri indicates a generally similar trend irrespective of the estimated length used in scaling the former. While length-standardized properties of Humerus III occasionally overlap with those in the observed Datong ranges, or more often fall below them, the properties of Humerus III usually overlap entirely with the observed lower half of the less robust Junziqing ranges of properties (i.e., between the observed group mean and minimum value), and only occasionally extend below them (Table S4).

The ranges of humeral length estimates for Zhoukoudian Humerus II and Humerus III fall in the upper half of the observed ranges for the Datong and Junziqing groups (Tables 1 and 2). The Tianyuan 1 humeral length also falls in the upper half of the observed Datong and Junziqing humeral length ranges (Table 1). This suggests that both recent modern Chinese groups may have been more small-bodied compared to other hominin groups in the sample, or at least appear to have had comparatively short (but still strong) humeri. Regardless of which may be the case, the ranges of differences exhibited by the two Zhoukoudian humeri fit within the lower half of the 2-fold or 3-fold greater range of observed length-standardized properties (i.e., maximum relative to minimum observed values) exhibited by these relatively numerically small groups of recent modern Chinese (Tables S4 and S5). This underscores the amount of variability that may be exhibited by recent modern humans, and provides quantitative support for the suggested modern-like aspects of Zhoukoudian humeral robusticity (Weidenreich, 1941).

## Discussion

This study demonstrates that East Asian *H. erectus* humeri (Zhoukoudian Humerus II and Humerus III) exhibit greater humeral rigidity and strength compared to an African *H. erectus* humerus (KNM-ER 1808). This difference exists whether one compares absolute values of properties, or properties scaled to the product of (averages of) estimated body mass and humeral length. Relative to humeri of Late Pleistocene hominins from Eurasia, the 1 Ma more recent *H. erectus* humeri from Zhoukoudian were consistently closer in robusticity than the older *H. erectus* humerus, KNM-ER 1808. While we could not acquire cross sections from Humerus II and Humerus III in the precise diaphyseal location as acquired from KNM-ER 1808 (i.e., an estimated 40% length location), a second location in Zhoukoudian humeri that was distal to midshaft, and also that avoided the deltoid tuberosity altogether, substantiated the midshaft comparisons. Support for comparisons between the different diaphyseal locations in the present study also comes from other studies (Sládek et al., 2010; Davies & Stock, 2014; Shaw et al., 2014; Mongle, Wallace & Grine, 2015a; Mongle, Wallace & Grine, 2015b) that report general similarities between mid-diaphyseal cross-sectional properties in human humeral or femoral cross sections sampled up to 20% length apart, and that have shown mid-diaphyseal cross-sectional properties differ trivially in cross sections that are approximately 5% length apart. Interestingly, the observed differences in diaphyseal robusticity documented in the present study occurred despite similar cortical thicknesses in KNM-ER 1808 and Humerus II, and a noticeably less thick diaphysis in Humerus III. This indicates that the greater subperiosteal areas of Zhoukoudian humeri (i.e., periosteal expansion) were more impactful on the observed robusticity differences compared to the more markedly different cortical thicknesses.

In considering the observed humeral robusticity differences of East Asian and African *H. erectus*, a few factors warrant further discussion. The approximate 1 Ma difference between the older African and more recent East Asian *H. erectus* humeri investigated in this study may reflect temporal evolutionary trends within the taxon (apart from general body size increases) in addition to any potential regional difference in body proportions or activity levels. Indeed, subsequent to the discovery of KNM-ER 1808, some have proposed reassigning African *H. erectus* material to a new taxon, *H. ergaster*, reflecting what is considered to be a different adaptive niche altogether (Wood, 1994). Postcranial evidence weighing in on the proposed adaptive differences between *H. ergaster* and *H. erectus* is sparse, however, and so the current study hopes to draw deserved attention to this critical issue. Discovery of contemporary *H. ergaster*/*H. erectus* humeri in Africa and East Asia would shed more definitive light on the matter, as could comparisons with additional *H. erectus* humeri from other geographic regions (e.g., West Asia and Southeast Asia). In the interim, it is worthwhile to consider potential differences in body proportions across individuals from these regions since they may introduce a potential confound in comparisons of humeral robusticity. Latitudinal clines in body proportions (i.e., Allen’s rule) have been well-documented in extinct and extant hominins (Allen, 1877; Ruff, 1994; Holliday, 1997; Tilkens et al., 2007; Ruff, 2010). Specifically, equatorial human populations, such as those from Africa, tend to have more linear body shapes and longer limbs relative to body mass compared to human populations from higher latitudes (e.g., the recent modern Chinese populations investigated in the present study), although aspects of environmental quality (e.g., nutritional differences) may modulate the phenotypic expression of these differences to some extent (Katzmarzyk & Leonard, 1998; Bogin et al., 2002; Bogin & Varela-Silva, 2010). This ecomorphological trend may characterize hominin body plans at least as early as archaic *H. sapiens* from the Middle Pleistocene of different regions, including East Asia (Trinkaus et al., 1999; Ruff, 2002b; Rosenberg, Lü & Ruff, 2006). While a portion of the observed differences between the size-standardized properties of Humerus II and Humerus III versus KNM-ER 1808 ultimately may be attributable to overall differences in *H. ergaster*/*H. erectus* body size and limb proportions, such as would be manifested in humeral length, we attempted to control for this possibility by also incorporating estimates of body mass in these scaling factors. Thus, our estimates of comparative humeral robusticity in *H. ergaster*/*H. erectus* reflect rigidity or strength *after* controlling for potential differences in estimated body size and limb length of individuals.

In addition to these observed differences in humeral diaphyseal robusticity, diaphyseal shapes of Humerus II and Humerus III diverged from that of the humerus of KNM-ER 1808 (i.e., the latter exhibited comparatively more equivalent *I*_max_ and *I*_min_ values; Tables 1 and 2 and Fig. 2), possibly hinting at potential differences in upper limb use. Additional suitable adult *H. ergaster*/*H. erectus* humeri from both regions would be needed in order to rigorously investigate this possibility further. Involvement of the upper limb in activities associated with selective advantages for hominins, and thus those that could be potentially worth future investigation in order to contextualize the observed differences in humeral diaphyseal robusticity or shape, include projectile throwing (Roach et al., 2013; Roach & Richmond, 2015), throwing in general (Shaw & Stock, 2009; Warden et al., 2009), spear thrusting (Schmitt, Churchill & Hylander, 2003), stone tool manufacturing (Rolian, Lieberman & Zermeno, 2011; Williams, Gordon & Richmond, 2012; Key & Dunmore, 2015), and scraping (Shaw et al., 2012). While some (Roach et al., 2013; Roach & Richmond, 2015) have attributed morphological evidence of projectile throwing to *H. erectus* (e.g., low humeral torsion, a human-like laterally-oriented scapular glenoid, and a tall mobile waist), there is no documented evidence of projectile use or throwing at Zhoukoudian, Locality 1. Unimanual scraping tasks, such as hide preparation, have been argued to generate bilateral asymmetry in upper limb muscle activity (Shaw et al., 2012), making it notable that side scrapers are the most abundant artifact in the Locality 1 archaeological assemblage (Pei & Zhang, 1985; Zhang, 2004; Li et al., 2011). To date, however, experimental assessments of loading associated with stone tool use and manufacturing focus on the hand rather than the forearm or arm (Rolian, Lieberman & Zermeno, 2011; Williams, Gordon & Richmond, 2012; Key & Dunmore, 2015). The roles these activities, or others, may have in inducing the dramatic right-side dominant asymmetry observed in diaphyseal strength of Late Pleistocene hominins in general (Sládek et al., 2016; Sparacello et al., 2017), or the Late Pleistocene hominin, Tianyuan I, in particular (Shang et al., 2007; Shang & Trinkaus, 2010), also remain unclear. Thus, caution is warranted when assessing right and left humeri from Zhoukoudian for potential activity-related bilateral asymmetry.

While Weidenreich (1941) may have emphasized external surface comparisons in describing the ‘thicker’ Humerus II as modern human-like in its robusticity, quantitative evaluation of internal structure supports this assessment of its humeral robusticity. Evaluation of Humerus III further corroborates the suggested similarity. Despite relative cortical thicknesses of Humerus II and Humerus III (%CA) exceeding those of the majority of individuals in both recent modern Chinese groups investigated in the study, which themselves were characterized by comparatively robust but short humeri, comparatively expanded subperiosteal areas of the recent modern Chinese humeri appear to be responsible for their typically higher measures of length-standardized humeral robusticity.

In the Late Pleistocene of Southeast Asia, comparatively smaller body sizes and statures have been reported compared to contemporaneous regional populations from Africa and Europe (Shackelford, 2007). The comparatively short humeri of both recent modern Chinese groups (i.e., Datong and Junziqing) suggest that these populations also may have been relatively small-bodied, or at least that they were characterized by short humeri. Both recent modern Chinese groups exhibited length-standardized humeral robusticity (e.g., *sJ* or *sZ*_p_) that bracketed that of the Late Pleistocene Tianyuan 1 hominin either in the upper half (Jinziqing) or lower half (Datong) of their observed ranges. Body mass of Tianyuan 1 has been estimated as 85.1 kg (Shang & Trinkaus, 2010). Both recent modern Chinese groups also exhibited observed ranges of length-standardized humeral robusticity that broadly overlapped with those of individuals comprising the East Eurasian Late Upper Paleolithic group (i.e., Minatogawa and Tam Hang). Average estimated body mass for these individuals is 51.4 kg, with a range of 42.3 to 70.5 kg (Table 1). Assuming general equivalence, or even minimal divergence in body sizes, both recent modern Chinese groups appear to have been characterized by less dramatic declines in humeral robusticity from Late Pleistocene levels compared to what is typically observed in Holocene populations (Ruff et al., 1993; Trinkaus, Churchill & Ruff, 1994; Trinkaus, 1997; Ruff et al., 2015a).

There are a few limitations in the current study that bear mention. We used anatomical markers to identify diaphyseal locations in our East Asian *H. erectus* sample (e.g., distal-most border of deltoid insertion), as one often is resigned to relying upon when analysing fossils that do not preserve entire bone lengths. This may have resulted in a small amount of imprecision when comparing diaphyseal locations. We also had to estimate medullary cavity size and dimensions in Humerus II. While Weidenreich (1941: Fig. 58B) provided information on relative size of the cavity, this was only in a single dimension, so we had to assume similarity in overall form to that of Humerus III. Nonetheless, the periosteal border is more impactful on cross-sectional properties than the endosteal border, as the current study demonstrates. While we used a range of length estimates for Humerus II to standardize properties for Humerus III, reasonably similar external contours of both humeri (see Figs. 1 and 2) suggest that the actual length of Humerus III probably fell within or close to this range of values. We were unable to assess the degree of bilateral asymmetry expressed in Zhoukoudian *H. erectus* humeri, which is noteworthy since the left Humerus II consistently exceeded the right Humerus III in structural properties. This is opposite the trend typically expressed in Late Pleistocene hominins that preserve both humeri (e.g., consider Tianyuan 1), suggesting perhaps the Zhoukoudian humeri represent two individuals.

Variability in published body mass estimates of KNM-ER 1808 and its purported pathological condition also bear further mention in this discussion. A two-fold range of body mass estimates attributed to KNM-ER 1808 have been recently published: 38.5 kg to 79 kg (Will & Stock, 2015; Antón, Potts & Aiello, 2014; Grabowski et al., 2015). The comparatively low most recent estimate of body mass, 38.5 kg (Grabowski et al., 2015), which we incorporated in our conservative use of an average estimate, may be an underestimate due to the authors’ reliance on cadavers in generating the original regression estimation equation (see Ruff et al., in press). If this estimate were more in line with the other higher published estimates, it would only further accentuate the comparatively lower robusticity of the KNM-ER 1808 humerus observed here. Alternatively, even when using such a low estimate of body mass (i.e., one standard deviation below our average estimate), Humerus II still slightly exceeds KNM-ER 1808 in a few aspects of humeral robusticity (e.g., *sI*_max_, *sZ*_max_, and *sJ*). Ultimately, we believe the use of an average estimate of body mass was the most conservative approach. Ruff (2008) noted that reactive bone formation on diaphyseal surfaces of KNM-ER 1808 could be differentiated from the original periosteal borders, lending confidence to the accuracy of calculating structural properties from the humeral diaphysis. However, the extent to which the condition responsible for the reactive bone formation may have altered the activity profile of the individual remains unknown, although presumably upper limb activities would have been impacted less than lower limb activities due to less reactive bone formation on the former.

Finally, the observed length-standardized robusticity displayed by the recent modern Chinese groups (Datong and Junziqing) relies on their body size estimates not dramatically exceeding those of Late Pleistocene hominins in the region (e.g., individuals from Tianyuan Cave, Minatogawa, and Tam Hang). Smaller body sizes of the recent modern Chinese groups would only further enhance their relative humeral robusticity. While a broader regional study of East Asian Holocene populations is beyond the scope of the current study, such a study would be necessary to better understand whether the Datong and Junziqing may be representative of regional trends in humeral robusticity.

## Conclusions

Consistent differences were observed between the more robust humeri of East Asian *H. erectus* (Zhoukoudian Humerus II and Humerus III) compared to the less robust humerus of African *H. erectus* (KNM-ER 1808). Zhoukoudian Humerus II and Humerus III also resembled Late Pleistocene hominins in humeral robusticity to a greater extent than the 1 Ma older KNM-ER 1808 humerus. This indicates the presence of regional differences in *H. erectus* humeral structure, which may reflect temporal trends (e.g., between *H. ergaster* versus *H. erectus*), ecogeographic trends in body proportions, and/or potential activity-related differences. Contemporaneous *H. ergaster*/*H. erectus* fossils from each region could begin to help resolve these non-mutually exclusive possibilities. Two recent modern Chinese groups also exhibited increased or equivalent humeral robusticity compared to *H. erectus* (Zhoukoudian Humerus II and Humerus III) and Late Pleistocene hominins from Asia (Tianyuan Cave 1, Minatogawa, and Tam Hang). Thus, quantitative evaluation of internal humeral structure supports the original description by Weidenreich (1941) of modern human-like robusticity of the Zhoukoudian Humerus II based on its external surface. A similar investigation of Zhoukoudian Humerus III provides corroborating support.

##  Supplemental Information

10.7717/peerj.4279/supp-1 Supplemental Information 1 Extra text, figures, and tables are provided

## Additional Information and Declarations

### Data Availability

The following information was supplied regarding data availability: The raw data has been provided in Supplemental Information 1.
