# Supplementary material for: Morphology and structure of Homo erectus humeri from Zhoukoudian, Locality 1
Source: PeerJ. 2018 Jan 19;6:e4279. doi: 10.7717/peerj.4279 (PMC5777375; doi:10.7717/peerj.4279)
Supplement: Supplemental Information 1 [file peerj-06-4279-s001.docx]

**Supplementary Information**

**SI Text**

**Text S1.** *Description of Zhoukoudian partial humerus (PA 64, Humerus III)*

Humerus III is a grayish-black right humeral shaft fragment, retaining a well-preserved surface and exhibiting a number of anatomical details. It is unclear whether the external color is due to intentional burning, or some unknown diagenetic process (e.g., mineral staining), although it is worth noting that *Weidenreich (1938, 1941)* describes other hominin material from the site as burnt (e.g., Femur II). *Woo & Chia (1954)* suggested that Humerus III represents the middle-third of a diaphysis, which we can confirm based on our observed similarities between its retained morphology and overlapping regions of the mirrored composite Humerus II rendering (Fig. 1). However, while the midshaft region is preserved on Humerus III, not enough of its diaphysis is retained to allow us to similarly evaluate the distinctive presence of a Humerus II-like “heavy” proximal half with an “almost circular” external contour and a “slender” distal half with a “triangular” contour (*Weidenreich, 1941: 55*).

*Woo & Chia (1954)* noted that overall muscle attachment sites on the external surface are generally not rugose, nor is the shaft particularly robust (Fig. 1). We generally agree. Overall, the preserved surface of Humerus III does not recall to us the same level of rugosity as described by *Weidenreich (1941)* for the original Humerus II diaphysis, or as is evident on a cast of the composite reconstruction made by Weidenreich (see Fig. S2). Humerus III also has distinctly different cortical thickness and medullary cavity dimensions compared to those attributed to Humerus II (*Weidenreich, 1941*: Fig. 58) and estimated in the present study (see Fig. 2). While this could indicate that Humerus III is from a non-adult individual, we find this less likely based on external dimensions of its cross sections (Fig. 2). Rather, it would seem more likely to us that Humerus III may represent a less robust adult male compared to Humerus II, or perhaps an adult female.

The proximal break through the shaft of Humerus III is irregular and runs oblique to the longitudinal axis of the shaft (Fig. 1). It passes through the distal region of the deltoid tuberosity on its anterolateral surface, while posteriorly it extends further proximally. On the medial side of Humerus III, the break extends distally until reaching the mid-anterior surface and extends proximally until reaching the mid-posterior surface of the diaphysis. Along its distal-most extent, the proximal break runs transversely across the anterior surface of the diaphysis until reaching the lateral surface. Distal to this section of the break, the deltoid tuberosity continues for approximately 14 mm. Overall, the lateral side of the proximal break recalls the general appearance of its medial side. *Woo & Chia (1954)* suggested a section of the proximal break is fresh, implying excavation damage. The distal break through the shaft is also irregular and runs oblique to the longitudinal axis of the shaft (Fig. 1). Its form mirrors the proximal break in extending more distally on the anterior surface and more proximally on the posterior surface. The transverse section of the distal break, however, is on the posterior surface of Humerus III whereas the transverse section of the proximal break is on its anterior surface. Posteriorly, the distal break extends proximally until immediately beneath the level of the nutrient foramen, while it extends well distal to the nutrient foramen on the anterior surface.

The deltoid tuberosity of Humerus III does not exhibit the same double-ridged form with an intervening depression that *Weidenreich (1941)* describes for Humerus II. Rather, it is a single anterolaterally-facing swelling of the diaphysis without an intrusive depression. It creeps around the lateral side of the shaft until it is visible in posterior view. This uninterrupted form may be due to only its distal-most portion being preserved on Humerus III, as even the double-ridged structure of Humerus II converges into a single structure distally (*Weidenreich, 1941*). Alternatively, it may indicate comparatively less rugosity of the insertion site for *m. deltoideus* on Humerus III compared to Humerus II. This latter possibility would be consistent with the other noted differences between Humerus II and Humerus III in terms of general surface rugosity, cortical thickness, and medullary cavity dimensions.

The anterior surface of the Humerus III shaft is convexly rounded with a maximum point of curvature immediately lateral to its central region (Fig. 1). Medially, the anterior surface gently slopes towards a rounded border separating it from the posterior surface. Contiguous flatness of the anteromedial border is greater in Humerus III than that portrayed in Humerus II (*Weidenreich, 1941*). On the medial border of Humerus III, a nutrient foramen lies immediately beneath the confluence of two diverging ridges extending distal to it on either side. Were these not interrupted by the distal break, they would seem to continue distally as the anterior and posterior rims of the medial supracondylar ridge. The foramen opens superiorly into a shallow groove that extends proximally for a few millimeters in length along the shaft. There is no distinct rugosity indicating the location of the *m.* *brachialis* origin on Humerus III.

The posterior surface of the Humerus III diaphysis is less rounded than its anterior surface (Fig. 1). Superiorly, the posterior surface faces predominantly posteriorly, while inferiorly it transitions to facing comparatively more medially. Posterior and inferior to the deltoid tuberosity, a radial groove can be palpated, but not easily distinguished by eye. The radial groove continues proximally along the shaft until it is ultimately interrupted by the proximal break, while distally it gradually dissipates into the anterolateral surface. The inferior extent of the origin of the lateral head of *m. triceps brachii* appears as a subtly protruding triangular area on the posterior surface, measuring 6 mm in mediolateral breadth at its widest point. While this area is interrupted by the proximal break, its rounded edges converge distally into a single ridge. A visible ridge on the proximolateral surface, immediately inferior to the radial groove, appears to define the origin of the medial head of *m. triceps brachii*. The ridge courses proximomedially to distolaterally across the posterior surface. At the level of the nutrient foramen, the ridge is interrupted on the lateral surface by the distal break. Medially on the shaft, a comparatively subtler ridge follows a parallel course for 7 mm, becoming defined only in the distal half of the preserved shaft. This latter ridge demarcates what eventually would appear to become the lateral supracondylar ridge. Not enough of this ridge is preserved in Humerus III, however, to make a direct form comparison to the “sharply edged” ridge attributed to Humerus I (*Weidenreich, 1941: 57*).

**Text S2.** *Backgroud information on comparative samples*

In order to compare humeral structural properties of East Asian *Homo erectus* humeri to those of adult African *H. erectus*, we calculated humeral structural properties from a published cross section of the individual represented by KNM-ER 1808 (*Ruff, 2008: Fig. 1*). This individual has been described as exhibiting a pathological condition that affected its long bone surfaces (*Walker et al., 1982*). Suggested diagnoses have attributed the reactive bone formation to hypervitaminosis A (*Walker et al., 1982*), to a treponemal infection such as yaws (*Rothschild et al., 1995*), or to sickle cell anemia (*Jefferson, 2004*), while the most recent evaluation suggests that the original hypervitaminosis A diagnosis is more likely than the others, although even it cannot be fully corroborated (*Dolan, 2011*). Regardless, original periosteal surfaces of diaphyses were “clearly distinguishable” from the pathological reactive bone deposits, which were also much thinner on humeri compared to those on femora (*Walker et al., 1982; Ruff, 2008*).

To provide a more informative contextual framework for evaluating Zhoukoudian humeral robusticity, we also compared structural properties from several Late Pleistocene Asian humeri. Levels of humeral robusticity expressed in Late Pleistocene hominins are traditionally higher than those expressed in Holocene modern humans (*Trinkaus et al., 1994; Sparacello et al., 2017*). Our comparative Late Pleistocene sample included partial right and left humeri of an adult skeleton (Tianyuan 1) recently discovered at the Chinese site of Tianyuan Cave (*Tong et al., 2004*). Accelerator mass spectrometry (AMS) and U-series dating on mammal teeth, and AMS radiocarbon dating on a hominin femur from Tianyuan 1, suggest that the hominin occupation at Tianyuan Cave occurred between 39-42 ka (*Shang et al., 2007; Shang & Trinkaus, 2010*). Systematic study of Tianyuan 1 has noted that it generally exhibited a mixture of features characteristic of early modern humans (e.g., crural indices) and features commonly observed among archaic humans (e.g., pronounced humeral midshaft asymmetry and tibial robusticity, the latter suggesting an emphasis on mobility; *Shang et al., 2007; Shang & Trinkaus, 2010*). Because of pronounced humeral asymmetry in Late Pleistocene hominins from multiple regions of the world (*Sládek et al., 2016; Sparacello et al., 2017*), and because the Zhoukoudian *H. erectus* sample included right and left humeri, we report separate right and left humeral properties of Tianyuan 1 and emphasize same-side comparisons when possible.

We also compiled published humeral cross-sectional data for additional Late Pleistocene Asian hominins. Specifically, we compared Late Upper Paleolithic modern humans (n = 10) from Minatogawa (individuals 1, 2, 3, 4) and Tam Hang (individuals 2, 3, 7, 11, 13, 14) (see *Sparacello et al., 2017*). The Minatogawa Fissure site is on the island of Okinawa, with charcoal fragments reportedly found near the skeletal material providing two radiocarbon (^14^C) dates of approximately 18 ka (*Baba & Narasaki, 1991; Baba et al., 1998; Kaifu et al., 2011; Matsu’ura & Kondo, 2011*). The Tam Hang rockshelter in Northern Laos has been radiocarbon (^14^C) dated to 15.7 ka (*Shackelford & Demeter, 2012*). Tam Hang occupies an inland location and its archaeological assemblage is characteristic of the regional Hoabinhian techno-complex (*Patole-Edoumba et al., 2015*). Lower limb diaphyses of the human skeletons recovered from Tam Hang appear to be generally gracile in comparison to other Late Pleistocene hominins from East and Southeast Asia (*Shackelford, 2007*).

Finally, to further establish the contextual framework for evaluating Zhoukoudian Humerus II and Humerus III, we compiled additional published Eurasian humeral data (*Churchill, 1994; Trinkaus et al., 1994; Trinkaus & Churchill, 1999; Crevecoeur 2008; Sparacello et al., 2017*) from Middle Paleolithic, Neanderthal, Early Upper Paleolithic and Late Upper Paleolithic hominins (see Table S1).

In addition to Late Pleistocene hominins, we acquired comparative structural properties from humeri of two recent modern Chinese populations. First, we sampled right humeri of adult individuals in the Datong population (n = 10), which lived in the Beiwei Dynasty during approximately the 5^th^ Century (*Han, 2005*). The Datong inhabited a basin surrounded by mountainous terrain with an elevation range of 500 to 2000 m. Nitrogen isotope data indicate a diet emphasizing meat consumption, and imply a pastoral subsistence strategy with little evidence of agriculturalism (*Zhang et al., 2010*). Sex attributed to individuals is unfortunately unavailable in this sample. Second, we sampled right humeri of adult individuals in the Junziqing population of the Qing Dynasty (1736-1851) (n = 23). This recently excavated material comes from Xinxiang City, Henan, central China. Xinxiang consists of plains, and historical records indicate that an agricultural economy was dominant during the Qing Dynasty. The Junziqing sample includes 11 females and 12 males, with sex determined according to traditional cranial and pelvic osteological indicators. The Junziqing had an agricultural subsistence strategy with no evidence of animal sacrifice, weaponry, or other relics that might be associated with stock raising or a hunter-gatherer lifestyle.

**Text S3.** *Deriving cross sections from Humerus II and Humerus III*

In order to estimate true cross-sectional properties from the diaphysis of Humerus II, we had to modify our approach. First, it is worth noting general similarity between the form of the external contour at our estimated Humerus II midshaft (Fig. 2) and the form of the external contour at Weidenreich’s estimated Humerus II midshaft (1941: Fig. 30 I). For Humerus II cross sections (Fig. 2), we estimated medullary cavity size using published descriptions and measurements of Humerus II (*Weidenreich, 1941*), as well as an anterior view radiograph of the original fossil (*Weidenreich, 1941: Fig. 58 B*). *Weidenreich (1941)* measured medullary canal breadth of Humerus II at its narrowest point (distal shaft) as 22% of transverse shaft diameter and 64% of the transverse shaft diameter at its widest point (proximal shaft). *Weidenreich (1941)* also described medullary canal dimensions as rapidly narrowing distal to the midshaft of Humerus II. Using *Weidenreich’s (1941: Fig. 58 B)* published radiograph of Humerus II, we measured medullary canal breadth as 36% of transverse shaft diameter at the same location where we estimated midshaft (Fig. S3). Our estimate of midshaft corresponded to a point slightly proximal to the beginning of the prominently constricted portion of the medullary canal in the radiograph, and just distal to the deltoid insertion on the external surface. Using a medullary cavity of the same general outline as that of Humerus III, we proportionately reduced the entire outline until its transverse breadth measured 36% of the transverse diameter of the Humerus II cast. This proportionately ‘reduced’ medullary cavity was centered in the transverse dimension of the Humerus II cast to produce the estimated cross section illustrated in Figure 2.

Because of the incomplete state of the partial right humerus from Zhoukoudian (Humerus III), locating comparable, fully intact cross sections from it also required a modified approach. We initially generated a mirrored rendering from the composite cast of the more complete left Humerus II reconstruction (*Weidenreich, 1941*), using the protocol outlined above. The mirrored rendering of the Humerus II composite cast was imported into VGStudio Max 2.1 (Volume Graphics GmbH, Heidelberg, Germany), and aligned following the procedure described above. The rendering generated from Humerus III was fit to the equivalent area of the aligned, mirrored rendering of the Humerus II composite cast using external features retained on both renderings (Fig. 1). While the original left Humerus II fossil did not retain proximal or distal articular ends, which were fully reconstructed in the composite cast produced by *Weidenreich (1941)*, the shaft region overlapping with Humerus III was fully intact. We estimated midshaft on Humerus III as the diaphyseal location immediately distal to the inferior edge of the deltoid tuberosity. On the more complete Humerus II composite reconstruction by *Weidenreich (1941)*, the analogous point immediately distal to the inferior edge of the deltoid tuberosity also generally approximated midshaft (see Figs 1, S1, and S3). In Datong and Junziqing comparative modern samples (n = 33), by comparison, all humeri exhibited a distal-most extent of the deltoid tuberosity between 53% and 43% of humeral length (where 0% length corresponds to the distal-most end of the diaphysis), while a majority exhibited even stronger correspondence between this anatomical landmark and midshaft (i.e., locations fell between 51% and 46% humeral length).

For both Humerus II and Humerus III (Fig. 2), we identified a second more distal location on diaphyses in an attempt to maximize comparability to the published 40% diaphyseal cross section of KNM-ER 1808, which itself was defined using an estimate of humeral length (*Ruff, 2008*). On Humerus III, we chose a second location 6.3 mm distal to its estimated midshaft location taking advantage of both anatomical landmarks that could be reliably identified (e.g., the most distal extent of the deltoid insertion and the nutrient foramen) and intactness of the cross section. More distal cross sections on Humerus III would have been incomplete due to missing parts of the fossil. By superimposing and aligning the Humerus II composite reconstruction (e.g., see Fig. 1), we defined an analogous location on Humerus II for its second more distal cross section. Using estimates of length for the composite reconstruction of Humerus II, this more distal location corresponds to approximately 48% diaphyseal length in the Zhoukoudian humeri. Following the same estimation procedure using *Weidenreich’s (1941: Fig. 58 D)* published radiograph of Humerus II as described above, we determined medullary cavity breadth of the more distal Humerus II cross section to be approximately 35% of its transverse external diameter (Fig. S3). This value (35%) was used to proportionately reduce a medullary cavity of the same general outline as that of Humerus III, which was centered in the transverse dimension of the Humerus II cast to produce the estimated cross section illustrated in Figure 2. Ultimately, while error in estimating true cross section locations on Zhoukoudian humeri may be present, it is worth noting that general similarities between mid-diaphyseal cross-sectional properties have been observed in human humeral and femoral cross sections sampled up to 20% length apart, while variability between mid-diaphyseal cross-sectional properties has been shown to be trivial in cross sections that are approximately 5% of length apart (*Sládek et al., 2010; Davies & Stock, 2014; Shaw et al., 2014; Mongle et al., 2015a, b*). Thus, given that sets of Zhoukoudian humeral cross sections are derived from approximately 50% and 48% diaphyseal lengths, and that both are distal to deltoid insertions, and that the 40% diaphyseal length cross section of KNM-ER 1808 (*Ruff, 2008*) is based on an estimate of humeral length itself, we believe these structural comparisons can be reasonably made.

**Text S4.** *Estimation of maximum length of Zhoukoudian Humerus II*

In order to estimate maximum length of the composite reconstruction of Zhoukoudian Humerus II, we performed a regression analysis using the combined Datong (n=10) and Junziqing (n=23) humeral sample (total n = 33). Specifically, as outlined in Eq. 1, we regressed the distance between the distal-most extent of the deltoid tuberosity and the proximal margin of the olecranon fossa against maximum length of the humerus since both of these landmarks were preserved on the Zhoukoudian Humerus II composite reconstruction by Weidenreich (1941) (see Figs. S2 - S4),

Maximum length = (distance between DT and PM)(1.544) + (133.172) Eq. 1

where DT = deltoid tuberosity and PM = proximal margin of olecranon fossa. The regression model is statistically significant (p < 0.001; R-squared = 0.551; Table S2), meaning the distance between these two landmarks is a statistically significant predictor of maximum length in the combined sample (n=33). Using this equation, we arrive at a predicted maximum length for the Zhoukoudian Humerus II composite reconstruction of 307.4 mm, where

307.4 mm = ((112.8235 mm)(1.544)) + (133.172)

**SI Figures**


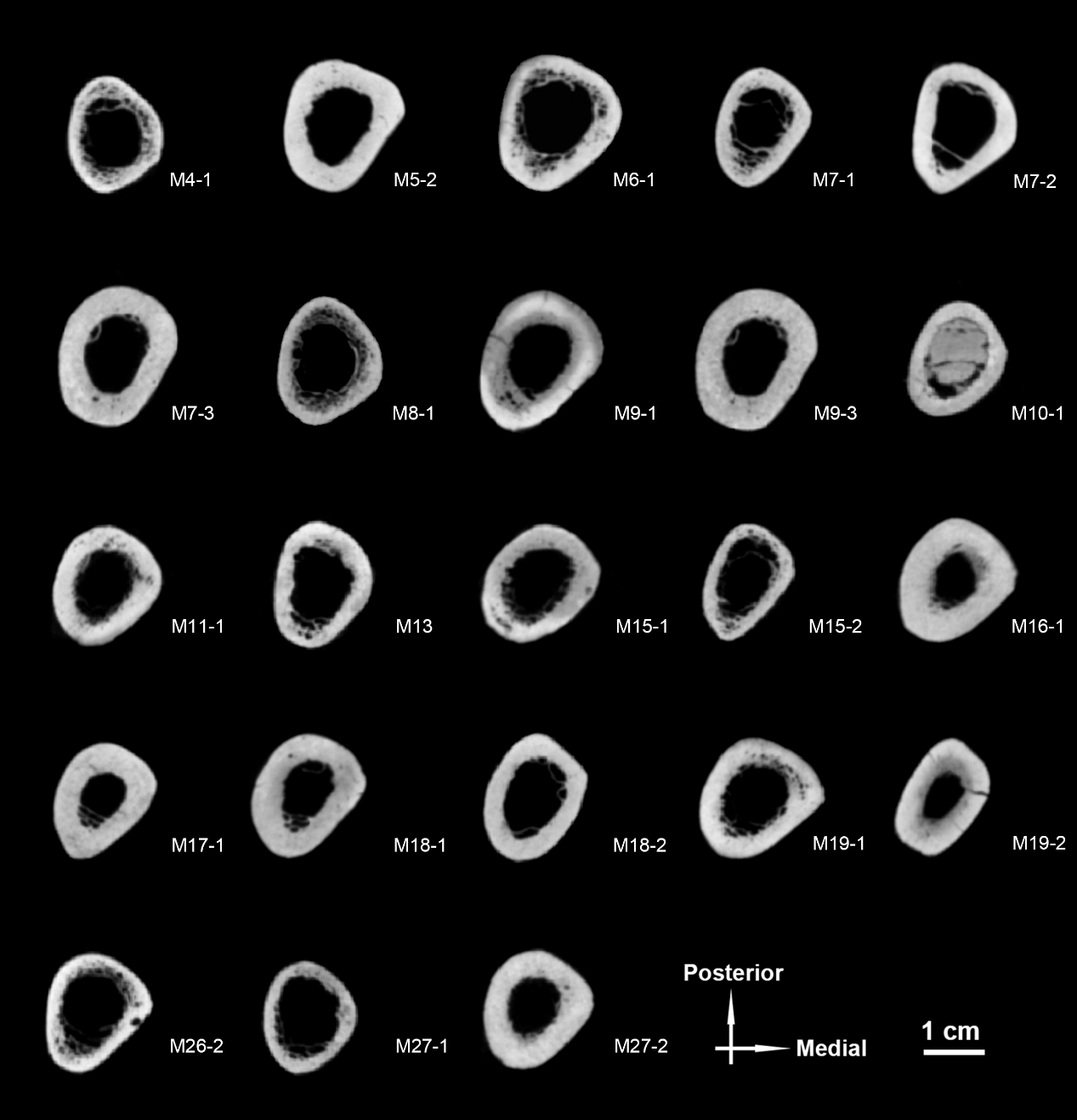


**Figure S1**. Humeral midshaft cross sections from the Junziqing population (n = 23).


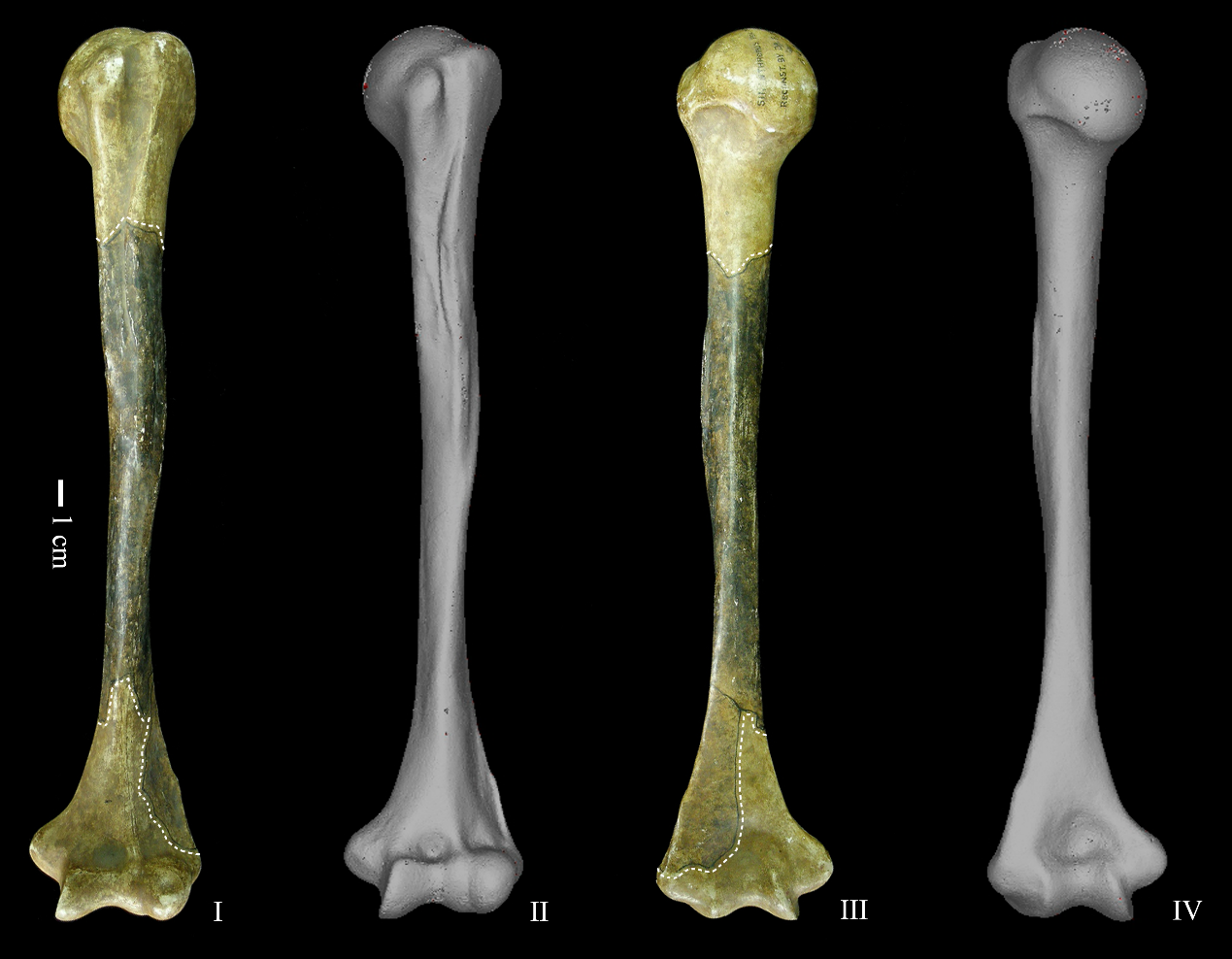


**Figure S2**. Anterior (I: cast, II: rendering) and posterior views (III: cast, IV: rendering) of the reconstructed composite Zhoukoudian left humerus (Humerus II). The original fossil parts of Humerus I and Humerus II are enclosed by dash lines. Note in image III that the proximal margin of the olecranon fossa is present.

**
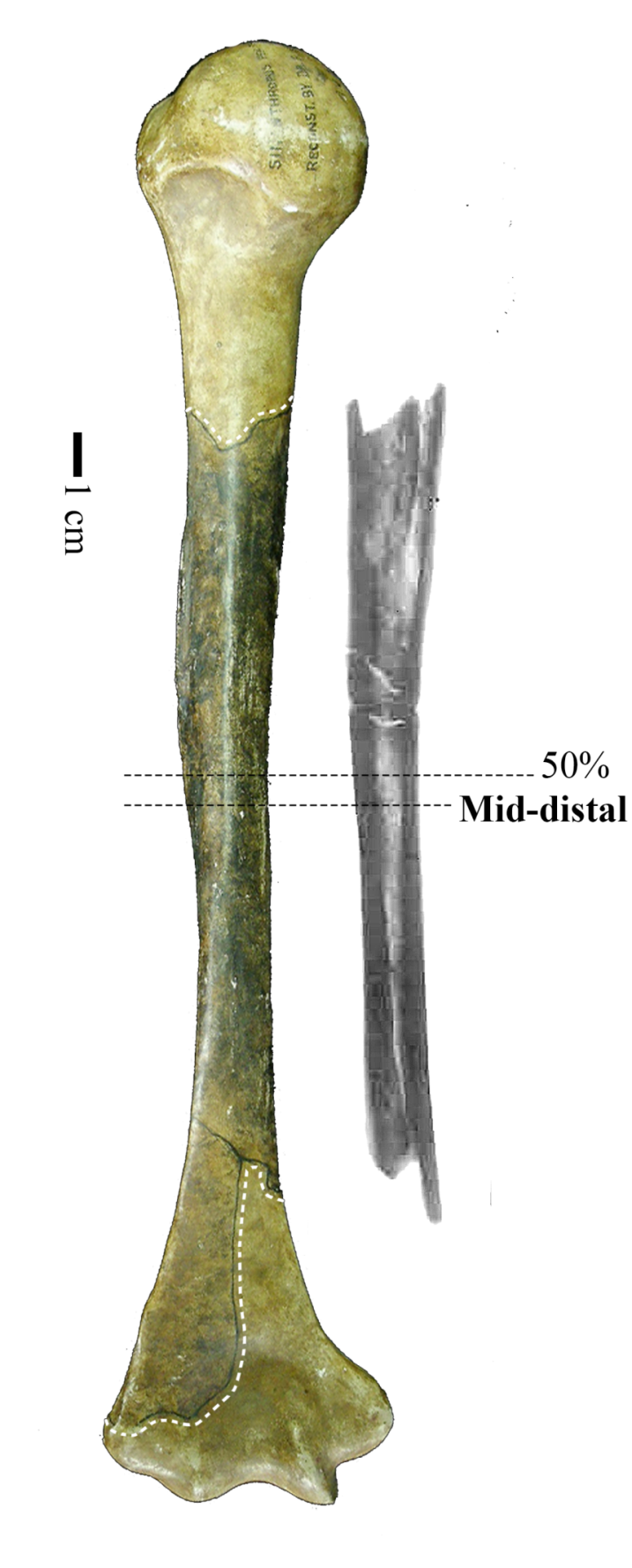
**

**Figure S3**. Posterior view of the Zhoukoudian left humerus (Humerus II) (left: composite cast made by Weidenreich; right: X-ray photograph of original Humerus II [Weidenreich, 1941: Figure 58 B]). The midshaft (50%) and more distal cross sections used in the present study are indicated by labelled dashed lines.


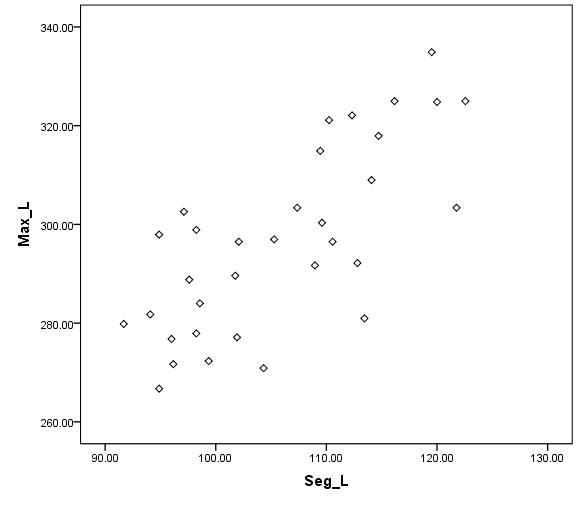


**Figure S4**. Bivariate plot of maximum length (Max_L) in mm and the distance between the distal-most extent of the deltoid tuberosity and the proximal-most margin of the olecranon fossa (Seg_L) in mm for the recent modern Chinese groups (n = 33).


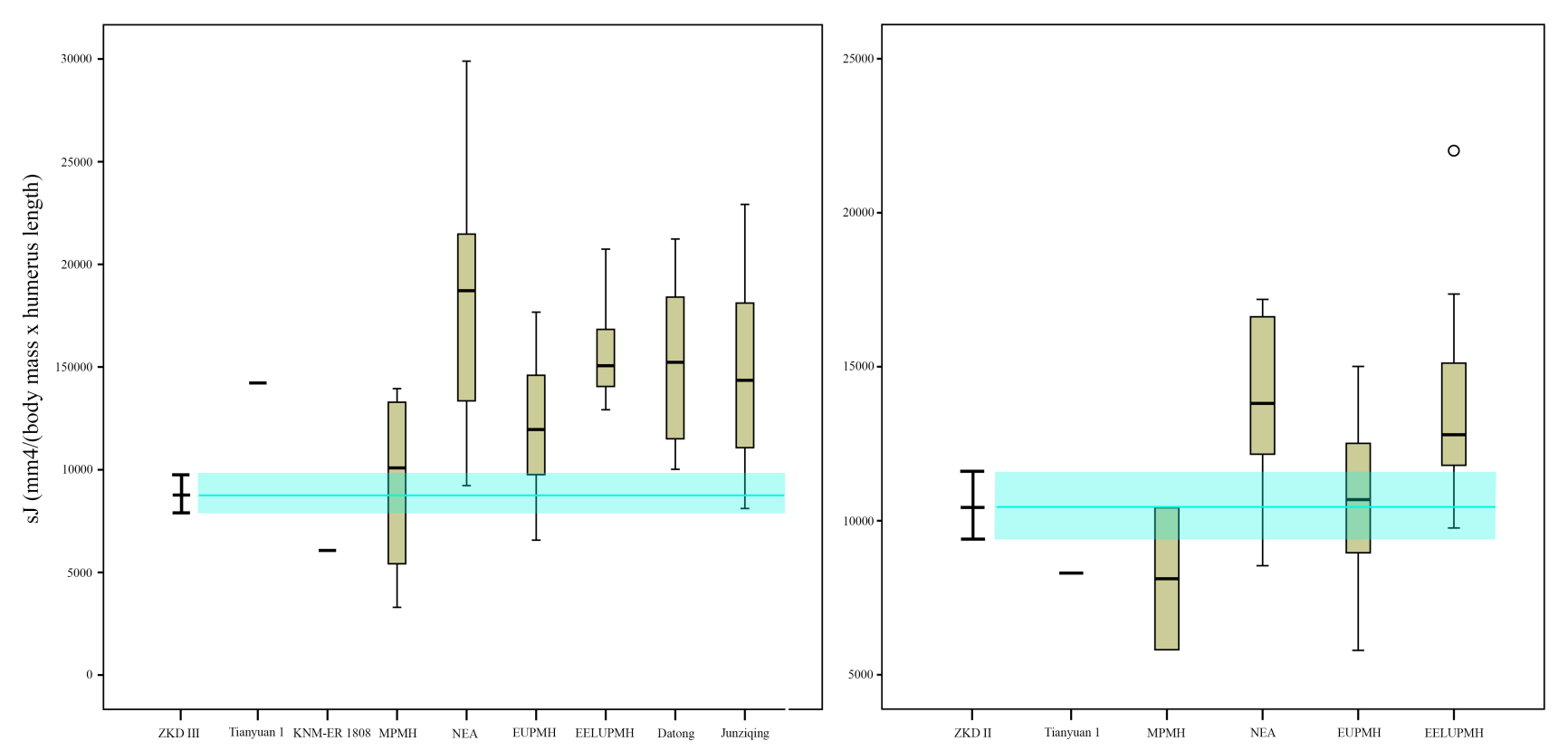


**Figure S5**. Box plots of midshaft torsional rigidity standardized to the product of estimated body mass and humeral length (sJ) of the Zhoukoudian right humerus (Humerus II and III) and comparative samples. The three different symbols, and corresponding cyan area and line, illustrated for Zhoukoudian Humerus II and III indicate the three different maximum lengths used in standardizing raw values. For Humerus II, Humerus III, and KNM-ER 1808, the average body mass estimate was used to standardize J. ZKD = Zhoukoudian; MPMH = Middle Paleolithic Modern Human; NEA = Neanderthal; EUPMH = Early Upper Paleolithic Modern Human; EELUPMH = East Eurasia Late Upper Paleolithic Modern Human.

**SI Tables**

**Table S1**. Comparative samples used in the present study.

|  | Right Humeri | Left Humeri | Data sources |
| --- | --- | --- | --- |
| African *H. erectus* | KNM-ER 1808(n=1) | - | Present study* |
| East Asian Late Pleistocene hominin | Tianyuan 1(n=1) | Tianyuan 1(n=1) | Present study |
| Middle Paleolithic Modern Human | Qfzeh 8, 9; Skhul 2, 5, 7 ( n=5) | Skhul 4, 5, 7 ( n=3) | *Trinkaus and Churchill, 1999*; *Sparacello et al., 2017* |
| Neanderthal | Kebara 2; La Chapelle 1; La Ferrassie 1, 2; La Quina 5; Lezetxiki 1; Oliveira 7; Palomas 92, 96; Regourdou 1; Shanidar 3, 4, 6; Spy 2; Tabun 1 (n=15) | Amud 1; Kebara 2; La Chapelle 1; La Ferrassie 1; La Quina 5; Palomas 16, 69; Spy 2; St.-Césaire 1 (n=9) | *Churchill, 1994; Trinkaus et al., 1994, Trinkaus and Churchill, 1999; Sparacello et al., 2017* |
| Early Upper Paleolithic Modern Human | Abri Pataud 3; Bausu da Ture 2; Caviglione 1; Cro-Magnon 1 (4294), 4296; Dolní Věstonice 3, 13, 14, 16; Grotte-des-Enfants 4; Khater 2; Mladeč 24; Nahal En'Gev 1; Nazlet Ostuni 12; Paglicci 25; Předmostí 3, 4, 9, 10, 14; Sunghir 1 (n=22) | Abri Pataud 3, 5; Barma Grande 5; Bausu da Ture 2; Cro-Magnon 4293; Dolní Věstonice 3, 13, 14, 16; Grotte-des-Enfants 4, 5; Mittlere Klause 1; Nazlet Khater 2; Ostuni 1; Paglicci 25; Pavlov 1; Paviland 1; Předmostí 3, 4, 9, 10, 14; Sunghir 1 (n=23) | *Churchill, 1994; Sparacello et al., 2017* |
| East Eurasia Late Upper Paleolithic Modern Human | Minatogawa 1, 2, 3, 4; Tam Hang 2, 3, 7, 11, 13, 14(n=10) | Minatogawa 1, 2, 3, 4; Tam Hang 2, 3, 7, 11, 13, 14(n=10) | *Kimura and Takahashi, 1992; Sparacello et al., 2017* |
| East Asian Holocene Modern Human | Datong, Shanxi Province (n=10) | - | Present study |
|  | Junziqing, Henan Province (n=23) | - | Present study |

*Properties calculated directly from the cross-sectional outline published by Ruff (2*008: Fig. 1*).

**Table S2.** Results of regression analysis for predicting humeral maximum length

| Model | Unstandardized  coefficient | | Standardized coefficient | t-statistic | p-value |
| --- | --- | --- | --- | --- | --- |
|  | β | Std. error | β |  |  |
| (Constant) | 133.172 | 26.637 |  | 5.00 | < 0.001 |
| Distance | 1.544 | 0.251 | 0.742 | 6.163 | < 0.001 |

**Table S3. Unstandardized and standardized properties of Zhoukoudian left humerus (II) and right humerus (III) at location distal to midshaft*.**

|  | Length  (mm) | Body mass  (kg) | TA  (mm^2^) | CA  (mm^2^) | %CA | Imax  (mm^4^) | Imin  (mm^4^) | Zmax  (mm^3^) | Zmin  (mm^3^) | J  (mm^4^) | Zp  (mm^3^) | sCA | sImax | sImin | sZmax | sZmin | sJ | sZp |
| --- | --- | --- | --- | --- | --- | --- | --- | --- | --- | --- | --- | --- | --- | --- | --- | --- | --- | --- |
| ZKD II^1^ | 307.4 | 53.6 | 253 | 214 | 84.6 | 6500 | 3852 | 608 | 489 | 10352 | 969 | 12.2 | 0.394 | 0.234 | 0.037 | 0.030 | 0.628 | 0.059 |
| **ZKD II^1^** | **315.7** | **53.6** | **253** | **214** | **84.6** | **6500** | **3852** | **608** | **489** | **10352** | **969** | **12.0** | **0.384** | **0.228** | **0.036** | **0.029** | **0.612** | **0.057** |
| ZKD II^1^ | 324 | 53.6 | 253 | 214 | 84.6 | 6500 | 3852 | 608 | 489 | 10352 | 969 | 11.9 | 0.374 | 0.222 | 0.035 | 0.028 | 0.596 | 0.056 |
| ZKD II^1^ | 307.4 | 55.3 | 253 | 214 | 84.6 | 6500 | 3852 | 608 | 489 | 10352 | 969 | 12.2 | 0.38 | 0.227 | 0.036 | 0.029 | 0.609 | 0.057 |
| ZKD II^1^ | 315.7 | 55.3 | 253 | 214 | 84.6 | 6500 | 3852 | 608 | 489 | 10352 | 969 | 12.0 | 0.372 | 0.221 | 0.035 | 0.028 | 0.593 | 0.056 |
| ZKD II^1^ | 324 | 55.3 | 253 | 214 | 84.6 | 6500 | 3852 | 608 | 489 | 10352 | 969 | 11.9 | 0.363 | 0.215 | 0.034 | 0.027 | 0.578 | 0.054 |
| ZKD II^1^ | 307.4 | 51.9 | 253 | 214 | 84.6 | 6500 | 3852 | 608 | 489 | 10352 | 969 | 12.2 | 0.407 | 0.241 | 0.038 | 0.031 | 0.649 | 0.061 |
| ZKD II^1^ | 315.7 | 51.9 | 253 | 214 | 84.6 | 6500 | 3852 | 608 | 489 | 10352 | 969 | 12.0 | 0.397 | 0.235 | 0.037 | 0.030 | 0.632 | 0.059 |
| ZKD II^1^ | 324 | 51.9 | 253 | 214 | 84.6 | 6500 | 3852 | 608 | 489 | 10352 | 969 | 11.9 | 0.387 | 0.229 | 0.036 | 0.029 | 0.616 | 0.058 |
| ZKD III^1^ | 307.4 | 53.6 | 268 | 178 | 66.4 | 6559 | 3962 | 639 | 465 | 10521 | 1009 | 10.2 | 0.398 | 0.240 | 0.039 | 0.028 | 0.639 | 0.061 |
| **ZKD III^1^** | **315.7** | **53.6** | **268** | **178** | **66.4** | **6559** | **3962** | **639** | **465** | **10521** | **1009** | **10.0** | **0.388** | **0.234** | **0.038** | **0.027** | **0.622** | **0.060** |
| ZKD III^1^ | 324 | 53.6 | 268 | 178 | 66.4 | 6559 | 3962 | 639 | 465 | 10521 | 1009 | 9.9 | 0.378 | 0.228 | 0.037 | 0.027 | 0.606 | 0.058 |
| ZKD III^1^ | 307.4 | 55.3 | 268 | 178 | 66.4 | 6559 | 3962 | 639 | 465 | 10521 | 1009 | 10.2 | 0.386 | 0.23 | 0.038 | 0.027 | 0.619 | 0.059 |
| ZKD III^1^ | 315.7 | 55.3 | 268 | 178 | 66.4 | 6559 | 3962 | 639 | 465 | 10521 | 1009 | 10.0 | 0.376 | 0.227 | 0.037 | 0.027 | 0.603 | 0.058 |
| ZKD III^1^ | 324 | 55.3 | 268 | 178 | 66.4 | 6559 | 3962 | 639 | 465 | 10521 | 1009 | 9.9 | 0.366 | 0.221 | 0.036 | 0.026 | 0.587 | 0.056 |
| ZKD III^1^ | 307.4 | 51.9 | 268 | 178 | 66.4 | 6559 | 3962 | 639 | 465 | 10521 | 1009 | 10.2 | 0.411 | 0.248 | 0.040 | 0.029 | 0.659 | 0.063 |
| ZKD III^1^ | 315.7 | 51.9 | 268 | 178 | 66.4 | 6559 | 3962 | 639 | 465 | 10521 | 1009 | 10.0 | 0.400 | 0.242 | 0.039 | 0.028 | 0.642 | 0.062 |
| ZKD III^1^ | 324 | 51.9 | 268 | 178 | 66.4 | 6559 | 3962 | 639 | 465 | 10521 | 1009 | 9.9 | 0.390 | 0.236 | 0.038 | 0.028 | 0.626 | 0.060 |
| **KNM-ER 1808^2^** | **350** | **60.2** | **240** | **197** | **82.1** | **5212** | **3891** | **503** | **457** | **9103** | **877** | **10.5** | **0.247** | **0.185** | **0.024** | **0.021** | **0.432** | **0.042** |
| KNM-ER 1808^2^ | 350 | 80.6 | 240 | 197 | 82.1 | 5212 | 3891 | 503 | 457 | 9103 | 877 | 10.5 | 0.185 | 0.138 | 0.018 | 0.016 | 0.323 | 0.031 |
| KNM-ER 1808^2^ | 350 | 39.8 | 240 | 197 | 82.1 | 5212 | 3891 | 503 | 457 | 9103 | 877 | 10.5 | 0.374 | 0.279 | 0.036 | 0.033 | 0.653 | 0.063 |

*Location was measured as 6.3 mm distal to the estimated midshaft on Humerus III. This location was chosen both because of its completeness and because it could be reliably located relative to preserved surface features (i.e., distal-most extent of the deltoid insertion and the nutrient foramen). An analogous location was used on the more complete Humerus II, which equated to an approximately 48% diaphyseal length location. Bold font indicates values standardized by average (length and) body mass estimates. ^1^Maximum length of the left Zhoukoudian Humerus II was reported by Weidenreich (1941) to be 324.0 mm. We estimated maximum length as 307.4 mm using a regression analysis of the distance between the deltoid tuberosity and the proximal margin of the olecranon fossa against maximum length on our comparative sample of Datong and Qing modern *Homo sapiens* (n = 33; see Text S4). In order to be conservative, we use both estimates to provide a range of standardized values for Zhoukoudian humeri about their average (315.7 mm). For Humerus II and Humerus III, we derived body mass estimates using the average (53.6 kg) of multivariate body mass estimates for Femur I (54.8 kg), Femur IV (54.3 kg), and Femur VI (51.6 kg) (Grabowski et al., 2015).^2^Cross-sectional data for an estimated 40% diaphyseal length section published by Ruff (2008). We used 350.0 mm for an estimated humeral length of KNM-ER 1808 (Ruff, 2008; pers. comm). For KNM-ER 1808, we derived an estimated body mass using the average (60.2 kg) +/- one standard deviation of three recently published estimates: 79 kg (Will and Stock, 2014), 63 kg (Antón et al., 2014: Table S2), and 38.5 kg (Grabowski et al., 2015).

**Table S4. Midshaft humeral length-standardized^1^ properties of Zhoukoudian right humerus (III) and comparative samples.**

|  |  | sTA | sCA | sI_max_ | sI_min_ | sZ_max_ | sZ_min_ | sJ | sZ_p_ |
| --- | --- | --- | --- | --- | --- | --- | --- | --- | --- |
| ZKD Humerus III | (307.4) | 26.5 | 17.7 | 6674 | 3704 | 1993 | 1429 | 10377 | 3012 |
|  | (315.7) | 25.1 | 16.8 | 5999 | 3329 | 1840 | 1319 | 9328 | 2781 |
|  | (324.0) | 23.8 | 15.9 | 5407 | 3001 | 1702 | 1220 | 8408 | 2573 |
| KNM-ER 1808 |  | 19.6 | 16.1 | 3473 | 2593 | 1173 | 1066 | 6066 | 2045 |
| Tianyuan 1 |  | 30.8 | 23.2 | 9192 | 5522 | 2599 | 1949 | 14714 | 3964 |
| Middle Paleolithic Modern Human^2^  (n=4) | Mean | 23.8 | 18.5 | 5659 | 3696 | - | - | 9355 | - |
|  | S.D. | 6.9 | 6.6 | 2961 | 1952 | - | - | 4891 | - |
|  | Min | 14.7 | 10.0 | 2138 | 1158 | - | - | 3297 | - |
|  | Max | 30.1 | 24.0 | 8345 | 5607 | - | - | 13952 | - |
| Neanderthal^2^  (n=11 for sTA, sCA, sI_x_, and sI_y_, sI_max_ and sI_min_; n=12 for sJ) | Mean | 34.6 | 26.7 | 11597 | 6734 | - | - | 18238 | - |
|  | S.D. | 7.2 | 6.7 | 3743 | 2686 | - | - | 6010 | - |
|  | Min | 25.0 | 15.4 | 6355 | 2868 | - | - | 9223 | - |
|  | Max | 45.8 | 39.3 | 18014 | 11887 | - | - | 29900 | - |
| Early Upper Paleolithic Modern Human^2^  (n=11 for sTA, sCA, sI_x_, and sI_y_; n=17 for sI_max_, sI_min_, and sJ) | Mean | 29.2 | 20.7 | 7227 | 4871 | - | - | 12098 | - |
|  | S.D. | 4.6 | 3.7 | 1909 | 1607 | - | - | 3383 | - |
|  | Min | 21.0 | 14.0 | 4315 | 2259 | - | - | 6574 | - |
|  | Max | 34.2 | 27.3 | 10396 | 8661 | - | - | 17671 | - |
| East Eurasia Late Upper Paleolithic^2^  (n=9) | Mean | 31.5 | 23.1 | 10178 | 5328 | - | - | 15506 | - |
|  | S.D. | 2.6 | 1.5 | 1651 | 976 | - | - | 2370 | - |
|  | Min | 28.3 | 21.7 | 8794 | 3895 | - | - | 12922 | - |
|  | Max | 36.9 | 26.9 | 13509 | 7228 | - | - | 20738 | - |
| Datong (n=10) | Mean  S.D.  Min  Max | 32.5  4.1  26.5  37.6 | 20.4  3.2  15.9  24.6 | 9394  2452  5877  12676 | 5811  1503  3939  7863 | 2514  501  1814  3128 | 1861  414  1365  2425 | 15206  3866  10035  20539 | 3871  793  2976  5004 |
| Junziqing (n=23) | Mean  S.D.  Min  Max | 31.1  3.9  24.4  41.7 | 18.6  4.2  11.3  27.1 | 8183  2309  4246  12413 | 5184  1535  2934  8899 | 2210  533  1287  3133 | 1763  401  1141  2481 | 13367  3753  7345  21312 | 3569  849  2220  5115 |

ZKD = Zhoukoudian ^1^Following others (Pearson, 2000; Carlson et al., 2007), areal properties (TA and CA) were standardized by dividing by humeral length^2^, then multiplying the result by 10^4^; section moduli (Z_max_, Z_min_, Z_p_) were standardized by dividing by humeral length^3^, then multiplying the result by 10^8^; principle moments of area (I_max_ and I_min_) and the polar moment of area (J) were standardized by dividing by humeral length^4^, then multiplying the result by 10^10^. ^2^Data from Churchill (1994), Trinkaus et al. (1994), Trinkaus and Churchill (1999), Crevecoeur (2008), and Sparacello et al. (2017). Humeral lengths used in standardizing properties are reported in Tables 1, except for ZKD humeri, where three different estimates were used (307.4, 315.7, and 324.0 mm) to standardize properties.

**Table S5. Midshaft humeral length-standardized^1^ properties of Zhoukoudian left humerus (II) and comparative samples.**

|  |  | sTA | sCA | sI_max_ | sI_min_ | sZ_max_ | sZ_min_ | sJ | sZ_p_ |
| --- | --- | --- | --- | --- | --- | --- | --- | --- | --- |
| ZKD Humerus II | (307.4) | 27.6 | 24.1 | 7823 | 4640 | 2203 | 1783 | 12462 | 3474 |
|  | (315.7) | 26.2 | 22.9 | 7032 | 4171 | 2034 | 1646 | 11203 | 3207 |
|  | (324.0) | 24.9 | 21.7 | 6338 | 3760 | 1882 | 1523 | 10098 | 2967 |
| Tianyuan 1 |  | 23.5 | 17.7 | 5162 | 3366 | 1718 | 1319 | 8528 | 2644 |
| Middle Paleolithic Modern Human^2^  (n=2) | Mean | 22.9 | 18.0 | 4725 | 3396 |  |  | 4725 |  |
|  | S.D. | 3.6 | 7.7 | 1714 | 1545 |  |  | 1714 |  |
|  | Min | 20.4 | 12.6 | 3513 | 2304 |  |  | 3513 |  |
|  | Max | 25.4 | 23.4 | 5937 | 4489 |  |  | 5937 |  |
| Neanderthal^2^  (n=4 for sTA, sCA, n=3 for sI_x_ and sI_y_; n=4 for sI_max_, sI_min_, and sJ) | Mean | 28.1 | 21.6 | 8904 | 4725 |  |  | 8904 |  |
|  | S.D. | 4.2 | 3.2 | 2443 | 1662 |  |  | 2443 |  |
|  | Min | 22.9 | 17.1 | 5847 | 2696 |  |  | 5847 |  |
|  | Max | 33.1 | 24.4 | 11321 | 6279 |  |  | 11321 |  |
| Early Upper Paleolithic Modern Human^2^  (n=14 for sTA, sCA, sI_x_, and sI_y_; n=19 for sI_max_, sI_min_, and sJ) | Mean | 28.0 | 18.9 | 6130 | 4093 |  |  | 6130 |  |
|  | S.D. | 3.9 | 2.9 | 1312 | 1005 |  |  | 1312 |  |
|  | Min | 21.3 | 14.2 | 3249 | 2450 |  |  | 3249 |  |
|  | Max | 37.1 | 24.6 | 8422 | 6173 |  |  | 8422 |  |
| East Eurasia Late Upper Paleolithic^2^  (n=7) | Mean  S.D.  Min  Max | 30.1  5.0  24.7  39.2 | 22.5  2.2  19.5  25.7 | 8902  2640  6010  13883 | 5157  1603  3759  8136 |  |  | 8902  2640  6010  13883 |  |

ZKD = Zhoukoudian ^1^Following others (Pearson, 2000; Carlson et al., 2007), areal properties (TA and CA) were standardized by dividing by humeral length^2^, then multiplying the result by 10^4^; section moduli (Z_max_, Z_min_, Z_p_) were standardized by dividing by humeral length^3^, then multiplying the result by 10^8^; principle moments of area (I_max_ and I_min_) and the polar moment of area (J) were standardized by dividing by humeral length^4^, then multiplying the result by 10^10^. ^2^Data from Churchill (1994), Trinkaus et al. (1994), Trinkaus and Churchill (1999), Crevecoeur (2008), and Sparacello et al. (2017). Humeral lengths used in standardizing properties are reported in Table 2, except for ZKD humeri, where three length values were used (307.4, 315.7, and 324 mm) to standardize properties.

**SI references**

**Antón SC, Potts R, Aiello LC. 2014.** Evolution of early *Homo*: an integrated biological perspective. *Science* **345**:1236828-1-1236828-13 DOI 10.1126/science.1236828.

**Baba H, Narasaki S. 1991.** Minatogawa Man, the oldest type of modern *H. sapiens* in East Asia. *The Quaternary Research* **30**: 221-230 DOI 10.4116/jaqua.30.22.

**Baba H, Narasaki S, Ohyama S. 1998.** Minatogawa hominid fossils and the evolution of Late Pleistocene humans in East Asia. *Anthropological Science* **106**:27-45 DOI 10.1537/ase.106.Supplement_27.

**Carlson KJ, Grine FE, Pearson OM. 2007.** Robusticity and sexual dimorphism in the postcranium of modern hunter-gatherers from Australia. *American Journal of Physical Anthropology* **134**: 9-23 DOI 10.1002/ajpa.20617.

**Churchill SE. 1994.** *Human upper body evolution in the Eurasian Later Pleistocene*. Ph.D. Dissertation, University of New Mexico.

**Crevecoeur I. 2008.** Étude anthropologique du squelette du Paléolithiquesupérieur de Nazlet Khater 2 (Égypte). Leuven University Press, Leuven, Belgium (318 p.).

**Davies TG, Stock JT. 2014.** Human variation in the periosteal geometry of the lower limb: signatures of behaviour among human Holocene populations. In: Carlson, K.J., Marchi, D., (Eds), *Reconstructing mobility – environmental, behavioral, and morphological determinants*. New York: Springer Press, 67-90.

**Dolan SG. 2011.** A critical examination of the bone pathology on KNM-ER 1808, a 1.6 million year old *Homo erectus* from Koobi Fora, Kenya. MA Thesis. New Mexico State University.

**Grabowski M, Hatala KG, Jungers WL, Richmond BG. 2015.** Body mass

estimates of hominin fossils and the evolution of human body size. *Journal of Human Evolution* **85**: 75-93 DOI 10.1016/j.jhevol.2015.05.005.

**Han W. 2005.** Analyses of the racial and systematic types of residents of Beiwei Period in Datong, Shanxi Province. *Research of China’s Frontier Archaeology* **4**: 270-280.

**Jefferson KL. 2004.** The possible selection of the sickle cell trait in early *Homo*. MS Thesis. Florida State University.

**Kaifu Y, Fujita M, Kono RT, Baba H. 2011.** Late Pleistocene modern human mandibles from the Minatogawa Fissure site, Okinawa, Japan: morphological affinities and implications for modern human dispersals in East Asia. *Anthropological Science* **119**: 137-157 DOI 10.1537/ase.090424.

**Kimura T, Takahashi H. 1992.** Cross sectional geometry of the Minatogawa limb

bones, 305–20. In: The Evolution and Dispersal of Modern Humans in Asia, ed. T. Akazawa, K. Aoki, and T. Kimura. Tokyo: Hokusen-sha

**Matsu’ura S, Kondo M. 2011.** Relative chronology of the Minatogawa and the Upper Minatogawa series of human remains from Okinawa Island, Japan. *Anthropological Science* **119**: 173-182 DOI 10.1537/ase.100322.

**Mongle CS, Wallace IJ, Grine FE. 2015a.** Cross-sectional structural variation relative to midshaft along hominin diaphyses. II. The forelimb. *American Journal of Physical Anthropology* **158**: 386-397 DOI 10.1002/ajpa.22802.

**Mongle CS, Wallace IJ, Grine FE. 2015b.** Cross-sectional structural variation relative to midshaft along hominin diaphyses. II. The hind limb. *American Journal of Physical Anthropology* **158**: 398-407 DOI 10.1002/ajpa.22802.

**Patole-Edoumba E, Duringer P, Richardin P, Shackelford L, Bacon A-M, Sayavongkhamdy T, Ponche J-L, Demeter F. 2015.** Evolution of the Hoabinhian Techno-Complex of Tam Hang Rockshelter in Northeastern Laos. *Archaeological Discovery* **3**:140-157 DOI 10.4236/ad.2015.34013.

**Pearson OM. 2000.** Activity, climate, and postcranial robusticity. *Current Anthropology* **41**: 569-609 DOI 10.1086/317382.

**Rothschild BM, Hershkovitz I, Rothschild C. 1995.** Origin of yaws in the Pleistocene. *Nature* **378**: 343-344 DOI 10.1038/378343b0.

**Ruff CB. 2008.** Femoral/humeral strength in early African *Homo erectus*. *Journal of Human Evolution* **54**:383-390 DOI 10.1016/j.jhevol.2007.09.001.

**Shackelford LL. 2007.** Regional variation in the postcranial robusticity of Late Upper Paleolithic humans. *American Journal of Physical Anthropology* **133**: 655-668 DOI 10.1002/ajpa.20567.

**Shackelford LL, Demeter F. 2012.** The place of Tam Hang in Southeast Asian human evolution. *Comptes Rendus Palevol* **11**: 97-115 DOI 10.1016/j.crpv.2011.07.002.

**Shang H, Tong H, Zhang S, Chen F, Trinkaus E. 2007.** An early modern human from Tianyuan Cave, Zhoukoudian, China. *Proceedings of the National Academy of Sciences of the United States of America* **104**: 6573-5678 DOI 10.1073/pnas.0702169104.

**Shang H, Trinkaus E. 2010.** *The early modern human from Tianyuan Cave, China*. College Station TX: Texas A&M University Press, College Station.

**Shaw CN, Stock JT, Davies TG, Ryan TM. 2014.** Does the distribution and variation in cortical bone along the lower limb diaphyses reflect selection for locomotor economy. In: Carlson, K.J., Marchi, D., (Eds), *Reconstructing mobility – environmental, behavioral, and morphological determinants*. New York: Springer Press, 49-66.

**Sládek V, Berner M, Galeta P, Friedl L, Kudrnová Š. 2010.** Technical note: the effect of midshaft location on the error ranges of femoral and tibial cross-sectional parameters. *American Journal of Physical Anthropology* **141**: 325-332 DOI 10.1002/ajpa.21153.

**Sládek V, Ruff CB, Berner M, Holt B, Niskanen M, Schuplerová E, Hora M. 2016.** The impact of subsistence changes on humeral bilateral asymmetry in Terminal Pleistocene and Holocene Europe. *Journal of Human Evolution* **92**: 37-49 DOI 10.1016/j.jhevol.2015.12.001.

**Sparacello VS, Villotte S, Shackelford LL. Trinkaus E. 2017.** Patterns of humeral asymmetry among Late Pleistocene humans. *Comptes Rendus Palevol* **16**: 680-689 DOI 10.1016/j.crpv.2016.09.001.

**Tong H, Shang H, Zhang S, Chen F. 2004.** A preliminary report on the newly found Tianyuan Cave, a Late Pleistocene human fossil site near Zhoukoudian. *Chinese Science Bulletin* **49**: 853-857.

**Trinkaus E, Churchill SE, 1999.** Diaphyseal cross-sectional geometry of Near Eastern Middle Palaeolithic humans: the humerus. *Journal of Archaeological Science* **26**: 173–184 DOI 10.1006/jasc.1998.0314.

**Trinkaus E, Churchill SE, Ruff CB. 1994.** Postcranial robusticity in *Homo*. II: humeral bilateral asymmetry and bone plasticity. *American Journal of Physical Anthropology* **93**: 1-34 DOI 10.1002/ajpa.1330930102.

**Walker A, Zimmerman MR, Leakey REF. 1982.** A possible case of hypervitaminosis A in *Homo erectus*. *Nature* **296**: 248-250 DOI 10.1038/296248a0.

**Weidenreich F. 1938.** Discovery of the femur and the humerus of *Sinanthropus pekinensis*. *Nature* **141**: 614-617 DOI 10.1038/141614a0

**Weidenreich F. 1941.** *The extremity bones of* Sinanthropus pekinensis*,* Palaeontogia Sinica New Series D **5**: 1-82.

**Will M, Stock JT. 2015.** Spatial and temporal variation of body size among early *Homo*. *Journal of Human Evolution* **82**: 15-33 DOI 10.1016/j.jhevol.2015.02.009

**Woo J, Chia L. 1954.** New discoveries of *Sinanthropus pekinensis* in Choukoutien. *Acta Palaeontologica Sinica* **2**: 267-288.

**Zhang GW, Hu YW, Pei DM, Song GD, Wang CS. 2010.** Stable isotope analysis of human skeletons from the Datong Beiwei archaeological site (southern suburbs). *Cultural relics in Southern China* **1**: 127-131.
